# Automatic calculation of symmetry-adapted tensors under spin-group symmetry: *STENSOR*, a new tool of the Bilbao Crystallographic Server

**DOI:** 10.1107/S1600576726000944

**Published:** 2026-02-27

**Authors:** Luis Elcoro, Jesus Etxebarria, J. Manuel Perez-Mato, Emre S. Tasci

**Affiliations:** a Department of Physics, Faculty of Science and Technology, University of the Basque Country, UPV/EHU, Bilbao, Spain; b Faculty of Science and Technology, University of the Basque Country, UPV/EHU, Bilbao, Spain; c Department of Physics Engineering Hacettepe University 06800 Ankara Türkiye; Australian Nuclear Science and Technology Organisation, Lucas Heights, Australia

**Keywords:** spin point groups, magnetic point groups, symmetry-adapted tensors, spin–orbit coupling, Bilbao Crystallographic Server

## Abstract

We present *STENSOR*, a new computational tool integrated into the Bilbao Crystallographic Server, designed for the automatic calculation of symmetry-adapted tensors under spin-group symmetry. The program requires either a file containing the structural data of the magnetic compound or the generators of the oriented spin point group, together with the generalized Jahn symbol associated with the tensor of interest. The user can propose any arbitrary tensor type or select a particular one from a predefined list. The program output returns the symmetry-adapted tensor under the spin point group and under the magnetic point group, which is also calculated. The comparison of these two tensor forms allows the coefficients due to spin–orbit coupling effects to be distinguished from those that have a non-relativistic origin and thus are usually more important. Two examples are given to illustrate the operation of the program.

## Abstract 2

*STENSOR* automatically computes symmetry-adapted tensors under spin and magnetic point groups. It generates tensors for both spin and magnetic point groups from structural data files or spin-group generators. The comparison permits separation of the effects due to spin–orbit coupling from non-relativistic contributions, as illustrated by examples.

## 1. Introduction

Spin groups describe the symmetries of magnetic materials in the non-relativistic limit, *i.e.* in the absence of spin–orbit coupling (SOC) (Litvin & Opechowski, 1974 ▸; Litvin, 1977 ▸; Liu *et al.*, 2022 ▸). Although they are approximate symmetries, spin groups have attracted considerable interest in recent years (Chen *et al.*, 2024 ▸; Jiang *et al.*, 2024 ▸; Xiao *et al.*, 2024 ▸), as they account for properties associated with physically significant effects. By contrast, effects arising from SOC are typically much weaker. Comparing the properties allowed by magnetic groups, which are exact symmetries, with those permitted by spin groups thus helps to distinguish features resulting from SOC from those that represent dominant, robust effects. For instance, when dealing with physical properties described by tensors, coefficients that originate from SOC vanish under spin-group symmetry but may remain nonzero under the corresponding magnetic group.

In this context, we have recently reviewed the transformation properties that the most important crystal tensors must satisfy under spin-group symmetry (Etxebarria *et al.*, 2025 ▸). By generalizing Neumann’s principle to spin point groups (SpPGs), we have determined the symmetry-adapted forms of key tensors describing equilibrium, transport and optical properties. We have found that tensor transformation rules under spin symmetry are significantly more varied and complex than those under magnetic symmetry.

Given a crystal whose symmetry is described by a specific spin space group (SpSG), the constraints that this symmetry imposes on a physical property represented by a tensor can be obtained by requiring the tensor to be invariant under the operations of the SpPG associated with the SpSG. The SpPG consists of operations of the form 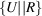, combining a spin part, represented by the matrix *U*, and a space part, represented by the matrix *R*. These matrices act on the components of a rank-*r* tensor *A*, transforming them in ways that depend on the nature of the tensor. In all cases, the transformation can be encoded through a combination of *r* labels V and M, together with the letters *e* and/or *a* in some cases (Etxebarria *et al.*, 2025 ▸), which characterize how each tensor component transforms under the action of *U* and *R*. These combinations generalize the Jahn symbols, commonly used for magnetic point groups (MPGs) (Jahn, 1949 ▸; Gallego *et al.*, 2019 ▸). Upon an operation 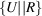 the transformation of the tensor involves the matrix *R* if the label is V and the matrix *U* for the M label. The letters *e* and *a* indicate, respectively, a change of sign in the transformation if *R* and *U* are improper operations. In other words, *e* and *a* denote that the tensor is even under space inversion and odd under time reversal, respectively. On some occasions square or curly brackets are included in the symbol, indicating symmetry or antisymmetry of pairs of indices. In the context of spin groups two contributions to the magnetization can be distinguished (Watanabe *et al.*, 2024 ▸; Etxebarria *et al.*, 2025 ▸), the spin contribution 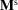, which is a rank-1 tensor of type M, and the orbital contribution 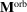, which is of type *ae*V. These symbols mean that 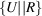 transforms the components of 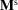 and 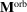 into 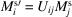 and 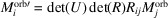, respectively (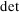 stands for determinant). Similarly, the spin contribution to the magnetoelectric tensor (inverse effect), which connects an applied electric field 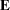 with an induced spin magnetization 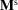 (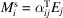), is of type MV, since an operation 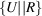 transforms vectors 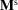 and 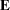 according to matrices *U* and *R*, respectively, and, therefore, the new 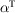 tensor must have components 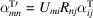. Analogously, a more complicated Jahn symbol such as *ae*MV{V^2^} means that 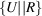 transforms a 4-rank tensor 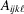 of components into 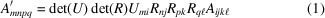
and is antisymmetric in the third and fourth indices. Here, all sub-indices range from 1 to 3, and all the components of 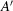, *A*, *R* and *U* are referred to the same orthonormal frame.

Etxebarria *et al.* (2025 ▸) list approximately 40 different generalized Jahn symbols to account for the most common tensor properties. Given this wide range of transformation behaviors, it seems desirable to have a tool capable of performing these calculations automatically. In this paper, we present such a tool, which we have named *STENSOR*, reflecting its clear parallelism with the program *MTENSOR*, used to obtain tensors adapted to the symmetry under MPGs (Gallego *et al.*, 2019 ▸). *STENSOR* is available through the Bilbao Crystallographic Server at the following address: https://cryst.ehu.eus/cryst/stensor.html.

A similar package for handling tensors that describe a few transport and optical processes has recently been reported by Xiao *et al.* (2025 ▸). The case of the second-order nonlinear conductivity tensor has been dealt with by Zhu *et al.* (2025 ▸).

## 2. The program

**Figure 1:**
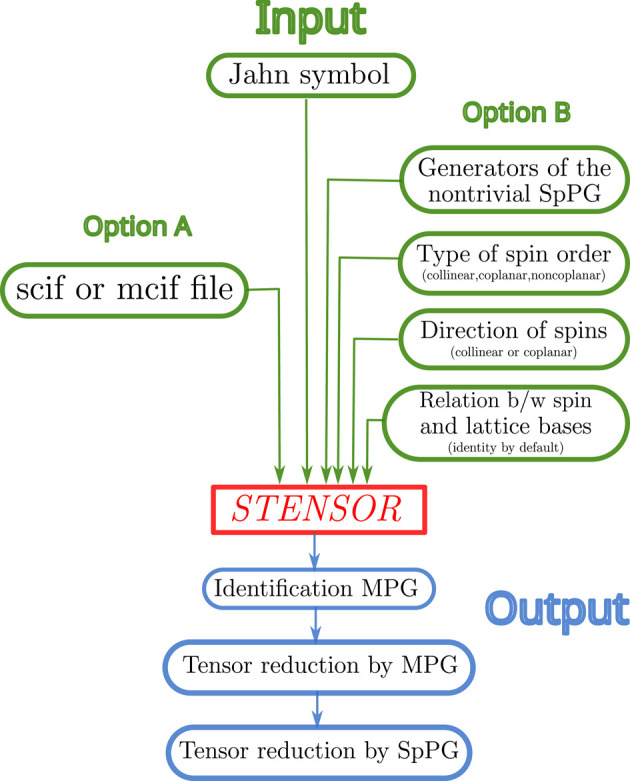
Flowchart of *STENSOR*. Apart from the Jahn symbol of the tensor that must be introduced in all cases, the input parameters (green color) are split into two different options. Whereas Option A needs just a scif or mcif file, in Option B the following parameters are required: (1) the generators of the nontrivial SpPG, (2) the type of spin ordering (collinear, coplanar, non-coplanar), (3) the direction of spins (collinear option) or the direction perpendicular to the plane of spins (coplanar option), and (4) the matrix that relates the basis in the spin space 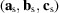 and the basis of the lattice 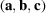. If this last matrix is not explicitly given the program assumes that it is the identity. The output of the program (blue color) gives (1) the identification of the MPG, (2) the reduced form of the tensor allowed by the MPG and (3) the reduced form of the tensor allowed by the SpPG.

*STENSOR* operates in a similar way to *MTENSOR*: given a specific tensor and a SpPG, the program returns the form of the tensor adapted to the symmetry of the specified group. The input data can be introduced in two alternative ways, referred to as Option A and Option B. In Option A, a file containing the structural data of the magnetic compound must be uploaded. Two file formats are supported: scif (spin cif) and mcif (magnetic cif). In Option B, the symmetry operations of the SpPG are entered manually. Fig. 1 ▸ shows a flowchart of the program.

**Figure 2:**
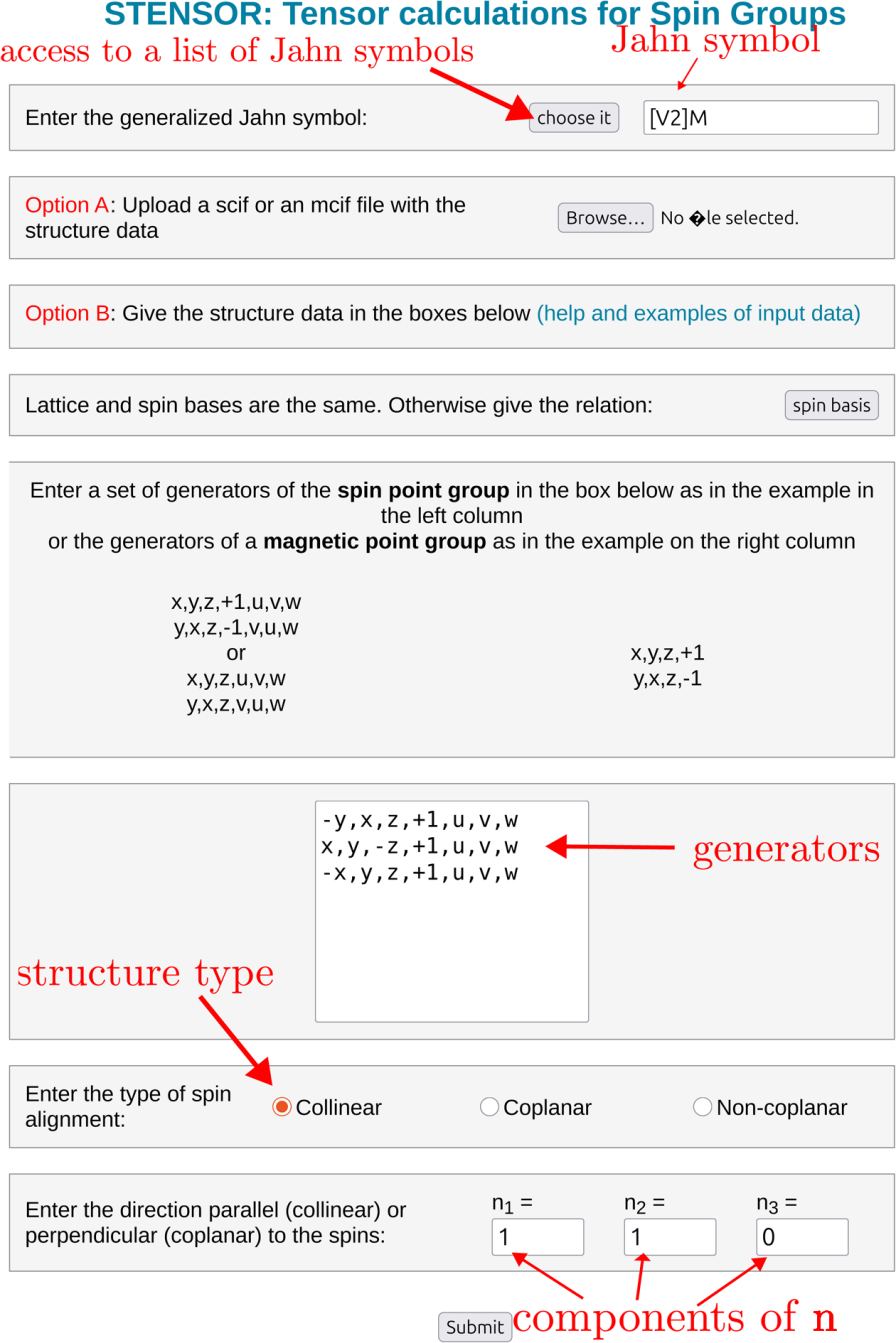
Input page of *STENSOR*. The parameters included correspond to the example of Section S2.

In both cases, the Jahn symbol corresponding to the tensor of interest must also be specified (top center of Fig. 2 ▸). *STENSOR* provides a list of the most common properties, including the corresponding generalized Jahn symbols and the constitutive equations that define the tensors. Nevertheless, in general, the user can construct any arbitrary Jahn symbol from scratch.

If Option A is selected and a scif file is provided, the input is already complete, since this file contains all the information needed to determine the symmetry-adapted tensor forms (in particular, all the symmetry operations of the SpPG are explicit). Alternatively, if an mcif file is uploaded, *STENSOR* assumes a minimal SpPG (Etxebarria *et al.*, 2025 ▸), *i.e.* a SpPG composed of the MPG operations together with possible spin-only operations characteristic of collinear and coplanar spin arrangements, whose presence is verified by the program. In this case as well, the input is complete. The format for scif files with magnetic structures described under their SpSGs is still under development, as an extension of the mcif format, where magnetic space groups (MSGs) are employed. A preliminary version of the scif format is already in use by the program *FINDSPINGROUP* (Chen *et al.*, 2024 ▸) and is supported by the latest version of *Jmol* (Hanson *et al.*, 2013 ▸). Integration of scif files into the MAGNDATA database (Gallego *et al.*, 2016 ▸) is planned for the near future.

In contrast, in Option B the SpPG operations are introduced manually by first giving a set of generators of the SpPG associated with the nontrivial SpSG as six-component vectors 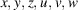 (symcard format). Note that the nontrivial SpPG involved in the calculation of the symmetry-adapted tensors is not restricted to one of the 598 groups listed by Litvin (1977 ▸). There are cases in which the nontrivial SpSG of the structure contains operations 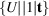 with 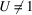 and 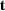 not being a lattice translation. In these cases, the operations 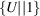 must be incorporated in the nontrivial SpPG and also contribute to the constraints of the tensor forms under the spin-group symmetry. The first three components refer to the lattice space 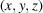 and the last three to the spin space 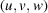. The lattice coordinates are expressed in a lattice basis 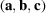 defined by the conventional unit cell and the axes normally used for the description of space operations. Thus, for example, trigonal and hexagonal lattices are always referred to an (oblique) hexagonal basis. The spin operations are in general defined in a different basis 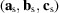, although, by default, they are referred to the same 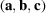 basis even in trigonal or hexagonal lattices. An option, however, exists to choose an independent spin reference frame by means of a transformation matrix. This option can be useful for example if the spin operations are described in an orthonormal reference system independently of the crystallographic frame, which is the convention adopted by many authors (Chen *et al.*, 2024 ▸; Jiang *et al.*, 2024 ▸; Xiao *et al.*, 2024 ▸). Alternatively to the six-component vector format, the generators can be introduced in a slightly modified format of seven-component vectors 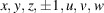, where the 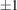 component is the determinant of the matrix that represents the operation in the spin space, 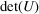. In this form, 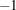 indicates explicitly that time reversal is included in the operation. Although this additional information is redundant, this format can be useful for obtaining more efficiently the transformation laws of tensors whose Jahn symbols do not contain the letter M. These cases can be described by means of an effective MPG and do not require the explicit form of the *U* operation, as only whether it is proper or improper is relevant.

A simpler possibility can also be used within Option B: instead of the previously described six- or seven-component vector format, one can input a set of generators of an MPG using the usual four-component vector format 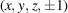. The program then interprets that the SpPG to be considered is obtained simply by adding to the MPG the spin-only symmetry associated with the collinearity or coplanarity of the spin arrangement. In a non-coplanar case, the assumed SpPG and the input MPG coincide. This format is particularly useful when working with minimal SpPGs, which statistically account for about 75% of magnetic structures (Gallego *et al.*, 2016 ▸; Chen *et al.*, 2024 ▸; Etxebarria *et al.*, 2025 ▸).

Once the SpPG associated with the nontrivial SpSG is known, the SpPG symmetry information must be completed by giving the type of magnetic structure (collinear, coplanar or non-coplanar), via the intrinsic spin-only group. This group is formed by elements of the form 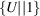, where *U* belongs to the groups 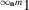, 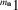 or 1 for the collinear, coplanar or non-coplanar cases, respectively. Here 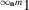 denotes the continuous group of all rotations around the direction of the spins together with all mirror planes containing this direction, while 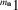 consists of the identity and a mirror plane with the orientation of the spin plane. In the collinear and coplanar cases the spin direction or the normal to the spin plane 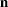 must be also provided. This direction is always given in the 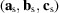 basis.

The output of the program consists of the following information:

(i) Setting used to express the space operations present in the SpPG, and crystal system. If a nonstandard setting is used, the transformation matrix to the standard setting is provided. The information for deducing the crystal system is extracted from the space-part operations of the SpPG.

(ii) Full set of symmetry operations of the nontrivial point group, deduced from the input generators.

(iii) Identified MPG as a subgroup of the SpPG, and the Jahn symbol of the tensor under the MPG symmetry. The MPG is recognized from the set of SpPG operations of the form 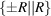, and its Jahn symbol can be easily derived from the symbol given for the SpPG by performing the substitution 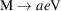 (Etxebarria *et al.*, 2025 ▸).

(iv) Complete form of the symmetry-adapted tensor under both the MPG and the SpPG. Depending on the space group of the space operations of the SpPG, different choices are possible for the reference frame used to express the tensor. In all cases an orthonormal frame is employed, which is also explicitly given.

## 3. Examples

We now present two examples of different complexity to illustrate key features of the program. The examples presented here were generated using Option B for the input data. Both examples correspond to real structures which have been taken from the MAGNDATA database (Gallego *et al.*, 2016 ▸). The first example extends the derivation of the piezomagnetic tensor for MnF_2_ by Etxebarria *et al.* (2025 ▸).

### 3.1. MnF_2_ (entry 0.15 in MAGNDATA)

**Figure 3:**
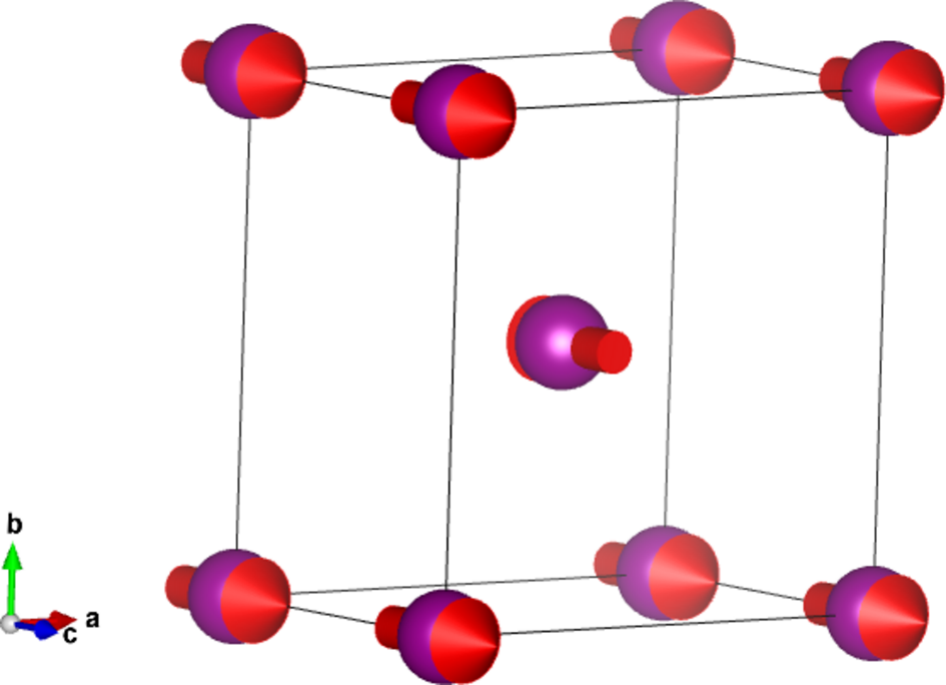
Magnetic structure of MnF_2_ showing only the magnetic (Mn) atoms.

The magnetic phase of MnF_2_ reported by Yamani *et al.* (2010 ▸) was assigned to the magnetic space group 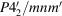 (No. 136.499) in MAGNDATA (Gallego *et al.*, 2016 ▸). The spin alignment presents a collinear structure with nontrivial SpPG 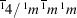, and spin orientation direction along [001] (see Fig. 3 ▸) (Chen *et al.*, 2024 ▸).

We choose the usual tetragonal crystallographic basis as the lattice and spin basis 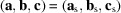. Under this setting, the generators of the nontrivial SpPG can be written as
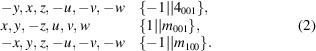
The right-hand column displays the symmetry operations in a generalized Seitz notation. The spin orientation is introduced by selecting ‘collinear’ along the [001] direction. For this spin orientation, the corresponding MPG is 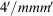. In this case, an alternative way to provide the input data using Option A is through the mcif file of the material since the SpPG is minimal. That file can be downloaded from MAGNDATA.

We now present some typical examples of tensor forms under the spin and magnetic symmetries. In all cases the tensors are expressed in an orthonormal basis with axes parallel to 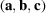. For example, considering a [V^2^]M tensor, which may represent the symmetric spin contribution 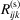 to the Hall effect resistivity, we obtain the following symmetry-adapted form for the MPG:
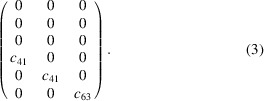
The Hall effect resistivity connects the electric field 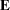 with the current density 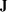 and the magnetic field 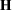 according to 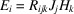. 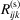 is the symmetric part of 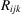, 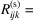

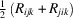
, and accounts for the linear magnetoresistance [see Grimmer (2017 ▸)]. In equation (3) we have used the Voigt notation, contracting the first two indices into a single index which ranges from 1 to 6. In contrast, only 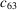 remains nonzero under the SpPG symmetry, which means that 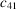 must be a small effect arising from SOC. Similarly, the antisymmetric part of the spin contribution to the Hall effect, 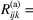

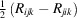
, which is of type *a*{V^2^}M, is entirely suppressed by the spin-group symmetry. However, under the MPG, two independent coefficients are allowed in the tensor, which is of the form 
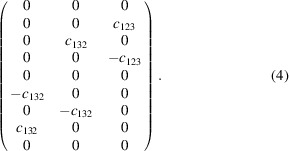
Therefore, in this case the entire property is expected to be a small relativistic effect.

Another example of a slightly different nature is the analysis of the spin splitting in energy bands. Among the predefined tensor properties available in *STENSOR* are the 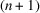-rank Cartesian tensors 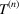, which describe the non-relativistic spin splitting of electronic bands and spin textures. According to Radaelli (2024 ▸), the direction of the spin electronic polarization and the magnitude of the non-relativistic spin splitting for a given band are given by the vector 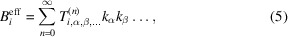
where 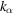 are the components of the wavevector, and the tensor coefficients depend on both the modulus of the wavevector and the band index. The Jahn symbols associated with 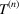 are M[V^*n*^] for even *n* and *a*M[V^*n*^] for odd *n*. For MnF_2_, it is straightforward to verify that the lowest-order nonzero tensor is 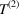, which contains a single surviving coefficient, 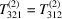. This result directly implies that the material is a *d*-wave altermagnet (Šmejkal *et al.*, 2022*a* ▸; Šmejkal *et al.*, 2022*b* ▸), exhibiting non-relativistic spin splitting proportional to 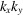 to lowest order. Furthermore, since 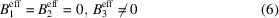
the electronic spin polarization is uniform to the lowest order across the entire Brillouin zone and is aligned with the 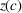 direction.

### 3.2. Mn_3_Sn (entry 0.199 in MAGNDATA)

**Figure 4:**
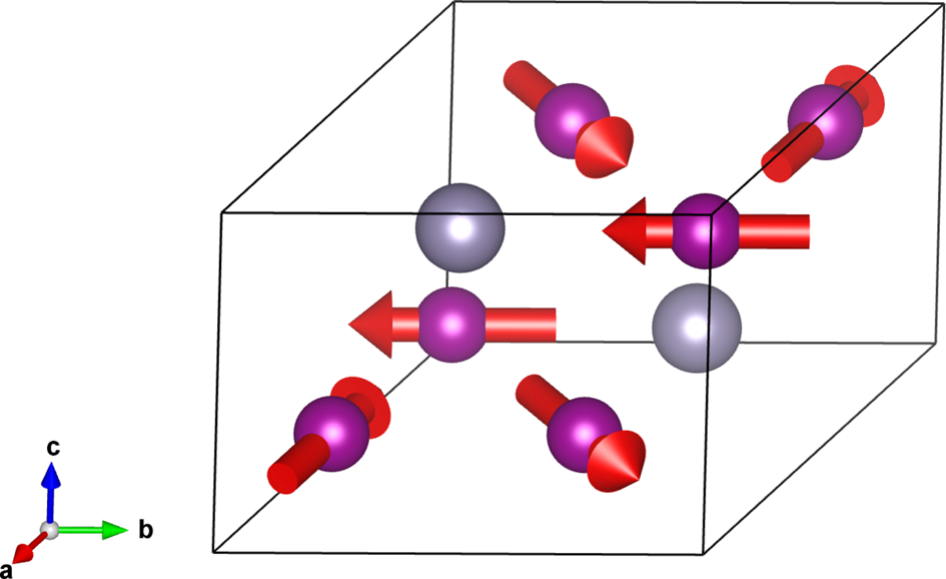
Magnetic structure of Mn_3_Sn showing the spins of the Mn atoms (entry 0.199 in MAGNDATA). Gray spheres represent the nonmagnetic Sn atoms. In the 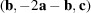 setting, the MPG is 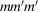.

One of the proposed structures for the magnetic phase of Mn_3_Sn has magnetic group 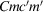 (No. 63.463) (Brown *et al.*, 1990 ▸) and, according to Chen *et al.* (2024 ▸), the SpPG is given by 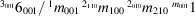, where the sub-indices refer to the hexagonal basis 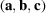 (see Fig. 4 ▸).

Initially, we use the same reference frame for both space and spin operations. The generators of the nontrivial SpPG can then be written as 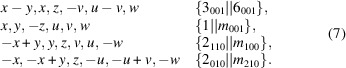
The type of spin alignment is in this case introduced in the program as ‘coplanar’, with the [001] direction perpendicular to the spin plane. Note that the ‘spin basis’ option has not been used so far, similar to the previous example, since 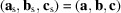.

We can now examine the shapes of different simple representative tensors. In all cases the tensors are expressed in an orthonormal basis with axes parallel to 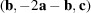. The MPG is identified as the 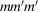 group in this same setting.

For example, an M-type tensor like the spin magnetization is null according to the SpPG but of the form 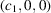 for the MPG symmetry, indicating that the material can show weak ferromagnetism of SOC origin along the first axis. Similarly, [V^2^] or [M^2^] tensors – which correspond to electric and magnetic susceptibilities (orbital contribution), or spin magnetic susceptibility – are diagonal uniaxial, 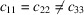, under the SpPG but diagonal biaxial under the MPG, 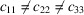.

Finally, the tensor describing the anomalous Hall effect (AHE, type *a*{V^2^}) is null for the SpPG, but results in
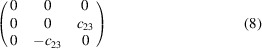
under the MPG, showing that a possible AHE in the material must be a SOC effect.

More complicated tensors can also be analyzed easily. For example, the spin contribution to the piezomagnetic tensor is of type M[V^2^]. Under the MPG that tensor is 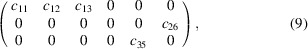
where we have used the contraction of the last two indices, while the SpPG symmetry gives 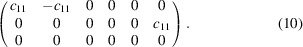
Since the 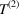 tensor for spin textures shares the same Jahn symbol as the spin contribution to the piezomagnetic tensor, and 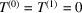 for Mn_3_Sn, equation (10) implies that the lowest-order non-relativistic spin splitting is described by the vector field 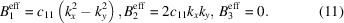
These relations indicate that this coplanar material also exhibits *d*-wave magnetism, with the electronic spin polarization confined to the atomic spin plane.

The orbital contribution to the piezomagnetic tensor has the same form (9) under the MPG but is null under the SpPG.

**Figure 5:**
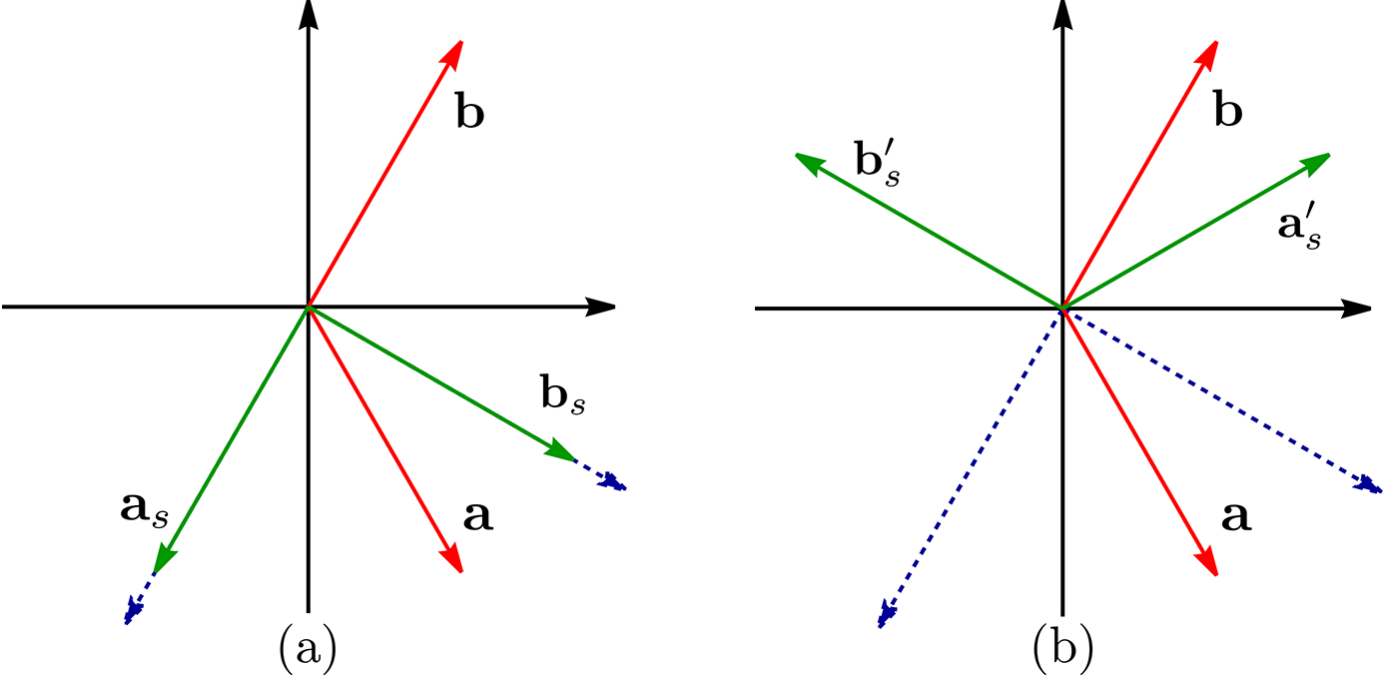
Relationship between the hexagonal unit vectors 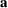 and 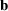 (red) of Mn_3_Sn and the spin basis vectors (green). In (*a*) a Cartesian basis, with vectors 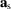 and 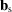, is used. In (*b*) an oblique spin basis, with vectors 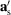 and 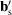, is employed to describe the Mn_3_Sn structure with the spins rotated by 90°. In both figures the blue dashed lines represent the first and second axes of the reference frame used to express the tensors.

We now illustrate the use of the ‘spin basis’ option by analyzing the same material but adopting this time a different reference frame for the spin space. For example, if we take an orthonormal spin basis given by [see Fig. 5 ▸(*a*)] 
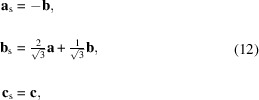
the generators can be expressed as 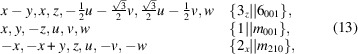
where the sub-indices *x* and *z* give the orientation of the two- and threefold axes with reference to the spin basis. Evidently, the same results are obtained in the end for the symmetry-adapted tensors. In the program, the required format for the square roots is sqrt(.), for both the basis relations [equations (12)] and operations [equations (13)].

**Figure 6:**
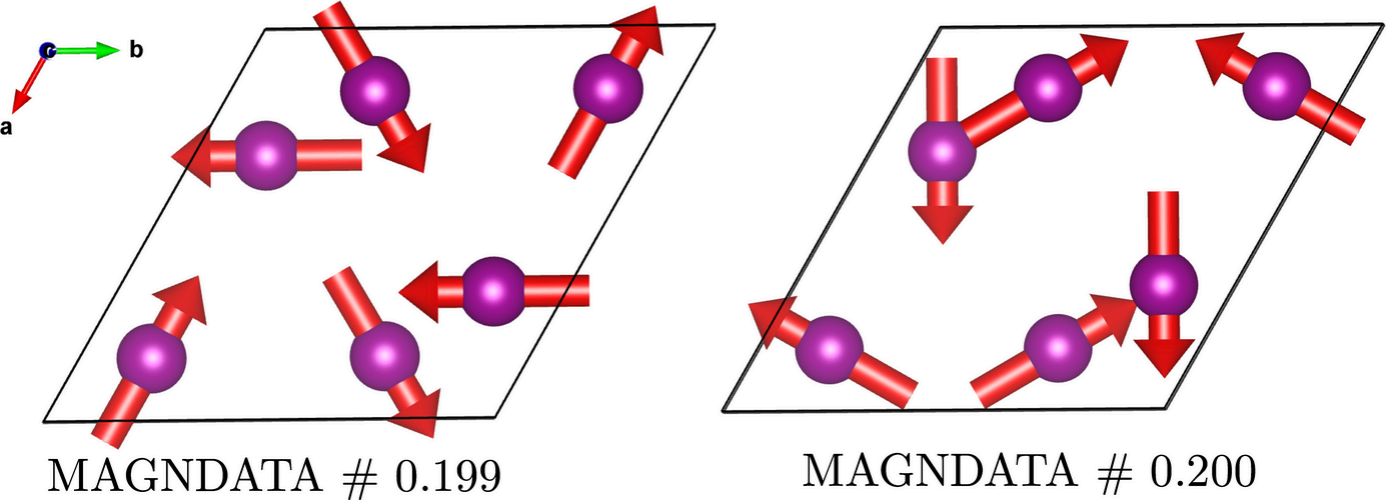
Two inequivalent structures of Mn_3_Sn showing only the spins of the magnetic atoms. The second structure is obtained from the first one by rotating the spins 90° counterclockwise about the *c* axis. In the 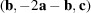 setting, the MPGs are 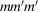 and 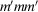, respectively.

The ‘spin basis’ option goes beyond the mere possibility of creating an orthogonal reference frame for the spin coordinates. In addition to the structure shown in Fig. 4 ▸, another model has been reported for Mn_3_Sn (Brown *et al.*, 1990 ▸), which experimentally could not be distinguished from the former. In this second model the spins are rotated by 90° counterclockwise around the *c* axis with respect to the spins in the first model (see Fig. 6 ▸). However, the two structures have different MSGs and are not physically equivalent. Thus, there are two different entries (0.199 and 0.200) in MAGNDATA. From the viewpoint of spin-group symmetry, the two structures have the same SpSG, as the only difference is a rotation of the spin arrangement. The spin operations *U* have, however, a different orientation with respect to the lattice, and this implies in general different tensor properties. Hence, the oriented SpPG of this second structure is 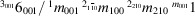 and, in this basis, the generators to be introduced for the nontrivial SpPG would be different. But, by using the spin basis option, we can work with the same input operations as in the previous case (0.199) and, instead, simply refer the spin operations of the input generators to a spin basis rotated by 90° around the *c* axis. The new spin basis is then [see Fig. 5 ▸(*b*)] 
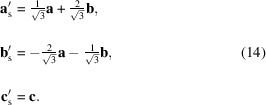
In this way, the former directions [110] and [010] in the spin space are now 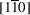 and [210], respectively, while the [001] direction remains invariant. Thus, the SpPG is correctly oriented for the structure of entry 0.200, with the corresponding MPG being now 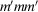. There are several instances where it can be seen that the rotated structure is not equivalent to the former structure. For example, it can be checked that the new symmetry allows weak ferromagnetism but with the magnetization along the second axis 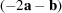 instead of the first one (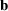), since an M-type tensor vanishes under the SpPG but takes the form 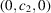 under the MPG. Similarly, equations (9) and (10) become 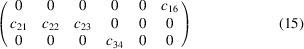 and 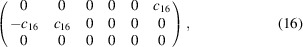
respectively, for the structure of entry 0.200. The change of the tensors can be rationalized as a mere 90° rotation of the spin degrees of freedom of the tensors with respect to the lattice. But, as directions differing by 90° in a hexagonal system are not equivalent, this difference is physically relevant.

## 4. Methods and technical details

*STENSOR* is a program written using *Mathematica* (Wolfram). Below, we provide a brief description of the mathematical algorithms used in the tensor reduction process, the general functioning of the program and several relevant technical details. Additional information can be found in the supporting information.

The program checks first that the input data introduced have a correct format. If Option A is used, it is assumed that the scif or mcif files are properly constructed. The program then simply verifies that the Jahn symbol is correct. In Option B, in addition to this check, the program confirms that the matrix relating the spin and space bases is non-singular, and that at least one of the three components 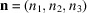 of the direction parallel (perpendicular) to the distributions of spins in collinear (coplanar) structures is nonzero. The program accepts floating-point numbers as components of 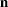, but they are immediately transformed as a set of integer numbers that represent the same direction. Every row in the box of generators must correspond to a pair of matrices 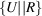 that are non-singular. Once the matrices have been constructed, it is checked that successive multiplications of the generators end in a finite number of symmetry operations so that the generated elements form a finite point group.

The calculation is divided into several steps, described in the following subsections.

### 4.1. Determination of the magnetic point group as a subgroup of the spin point group

Once all the operations 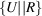 of the SpPG associated with a nontrivial SpSG have been determined, based on the scif or mcif files (Option A) or the introduced set of generators (Option B), those elements within the SpPG that also belong to the MPG are identified. Provided that the spin and lattice bases coincide, these elements are of the form 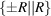. If the two bases differ, the identification is carried out by first performing a change of basis in the spin space.

As demonstrated in Section S1.1 of the supporting information, in the collinear and coplanar cases, an efficient algorithm to identify such operations is through the condition 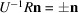. For non-coplanar groups the identification is straightforward. If an mcif file is used in Option A, a minimal SpPG is assumed and the MPG operations are explicit.

Once such operations are listed, the program determines the corresponding MPG. Since the operations are generally not expressed in the standard setting of the identified MPG (Litvin, 2013 ▸), *STENSOR* provides a transformation matrix *P* that changes the original setting to the standard one. This transformation satisfies the relation 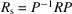, where 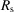 is the matrix representation of the symmetry operation in the standard setting.

### 4.2. Tensor reduction under the magnetic point group

The tensor reduction under the MPG is performed using projectors (Bradley & Cracknell, 1972 ▸; Dresselhaus *et al.*, 2008 ▸). The procedure is carried out in two steps. In the first step, possible symmetry or antisymmetry between pairs of tensor indices is not considered, and only the point-group operations are applied. In the second step, the additional constraints imposed by the (anti)symmetry of the tensor indices are incorporated. The procedure involves working with matrices 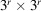, where *r* is the rank of the tensor to be reduced. To simplify the handling of these matrices *STENSOR* performs a row reduction (or Gauss decomposition) to convert them into the reduced row echelon forms. Additionally, the *SparseArray* functionalities implemented in *Mathematica* are used to efficiently store and operate on large matrices with a small fraction of nonzero elements. These options greatly improve the memory and CPU-time requirements in the calculations and are especially useful for finding the symmetry-adapted forms of tensors with large ranks. A detailed explanation of the method is provided in the supporting information (Sections S1.2.1, S1.2.2 and S1.2.3).

### 4.3. Tensor reduction under the spin point group

The calculation of the tensor reduction under the full spin group can be divided into three steps, with the first two being almost identical to those used for the reduction under the MPG. First, the constraints imposed by all operations of the SpPG, except those belonging to the intrinsic spin-only group, are determined. Next, the symmetry constraints arising from the symmetry or antisymmetry of the tensor indices are incorporated. Finally, as a third and final step, the additional reduction imposed by the intrinsic spin-only point group in collinear and coplanar spin arrangements is applied. Here, as well, the entire procedure employs the projector technique (except for the infinite-fold axis in the collinear spin-only group), Gaussian decomposition and the *SparseArray* method.

In the case of the collinear spin-only group, the order of the group 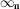 is infinite, and it is not possible to define a projector; therefore, a different procedure must be followed. This infinite group can be regarded as generated by an infinitesimal rotation about 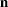 by an angle 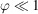. Expanding the matrix representing such a rotation as a series and retaining only the linear term, one can show that the invariance condition under 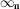 leads to a set of homogeneous linear equations. These equations impose linear relations among the independent tensor coefficients, thereby completing the tensor reduction. The entire procedure is thoroughly explained in Sections S1.3.1 and S1.3.2 of the supporting information. In addition, an explicit example illustrating all steps of the method in detail is provided in Section S2.

## 5. Related literature

The following references are cited only in the supporting information: Grimmer (1993 ▸), Nye (1985 ▸), Spaldin *et al.* (2008 ▸), Tomiyasu & Kagomiya (2004 ▸).

## 6. Conclusions

This study has presented *STENSOR*, a new addition to the Bilbao Crystallographic Server, which automates the derivation of symmetry-adapted tensor forms under SpPG symmetry. The tool can operate either from structural files (scif or mcif) or from manually provided generators of the oriented SpPG in combination with a generalized Jahn symbol, producing as output the tensor forms allowed by both the SpPG and MPG. This double output makes it straightforward to identify which tensor components have non-relativistic origin and which ones typically arise from SOC. From a computational point of view, *STENSOR* is implemented in *Mathematica*, and relies on algorithms such as the projector method, Gaussian reduction and *SparseArray* handling to reduce memory and CPU requirements. Tensors of ranks as high as 6 or 7 can be processed efficiently, with computation times remaining reasonable.

The case of two real materials, collinear MnF_2_ and coplanar Mn_3_Sn, has been analyzed to illustrate some of the *STENSOR* capabilities in determining the forms of representative tensors. In MnF_2_, *STENSOR* finds a *d*-wave altermagnetic spin splitting (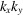 dependence) with uniform electronic spin polarization along the *z* axis. For Mn_3_Sn, *d*-wave magnetism is also identified, but here the spin polarization is confined to the *x*–*y* plane. In this compound, it is also shown how the spin-basis option can be used to describe structures that differ in a rigid spin rotation. *STENSOR* complements existing tools such as *MTENSOR* and databases like MAGNDATA within the Bilbao Crystallographic Server. The program is expected to be useful to distinguish SOC-driven versus non-relativistic effects and may facilitate systematic studies in magnetic compounds.

We conclude with a note of caution: experimental magnetic structures may incorporate features that originate from SOC, thereby reducing the actual SpPG symmetry with respect to the ideal SOC-free case. In such situations, the comparison of tensor forms under MPGs and SpPGs does not separate properly relativistic from non-relativistic contributions. In fact, tensor components allowed under the observed (reduced) SpPG may in practice require nonzero SOC.

## Supplementary Material

In the supporting information all the details of the used algorithm are explained. DOI: 10.1107/S1600576726000944/in5111sup1.pdf

## Data Availability

The program *STENSOR* is hosted in the *Magnetic Symmetry and Applications* section of the Bilbao Crystallographic Server (https://cryst.ehu.eus). It can also be accessed via the direct link https://cryst.ehu.eus/cryst/stensor.html.
