# Supplementary material for: Automatic calculation of symmetry-adapted tensors under spin-group symmetry: STENSOR, a new tool of the Bilbao Crystallographic Server
Source: J Appl Crystallogr. 2026 Feb 27;59(Pt 2):640–7. doi: 10.1107/S1600576726000944 (PMC13060620; doi:10.1107/S1600576726000944)
Supplement: Supplementary file 1 [file j-59-00640-sup1.pdf]

JOURNAL OF APPLIED CRYSTALLOGRAPHY

Volume 59 (2026)

Supporting information for article:

Automatic calculation of symmetry-adapted tensors under spin-group symmetry: *STENSOR*, a new tool of the *Bilbao Crystallographic Server*

Luis Elcoro, Jesus Etxebarria, J. Manuel Perez-Mato and Emre S. Tasci

Supplementary Material of ”Automatic calculation of symmetry-adapted tensors under spin-group symmetry. ***STENSOR***, a new tool of the ***Bilbao Crystallographic Server*´.”**

Contents of Supplementary Material

S1 Methods and technical details **2**

S1.1 Determination of the magnetic point group as a subgroup of the spin point group . . 3

S1.2 Tensor reduction under the magnetic point group 4

S1.2.1 Tensor reduction by the operations of the MPG 4

S1.2.2 Further tensor reduction under (anti)symmetrization of indices 6

S1.2.3 Some considerations on the algorithm 8

S1.3 Tensor reduction under the spin point group 10

S1.3.1 Tensor reduction by the operations of the SpPG 11

S1.3.2 Tensor reduction by the operations of the trivial group 12

S1.3.3 Symmetry constraints of tensors that contain toroidic components 14

S2 Example: symmetric spin contribution to the Hall effect in $NiCr_2O_4$ **15**

S2.1 Determination of the MPG 16

S2.2 Tensor reduction by the MPG 16

S2.3 Tensor reduction by the SpPG 18

S1 Methods and technical details

In Option A, all the required data except the Jahn symbol are obtained from the uploaded scif or mcif files. In a scif file the symmetry operations have the form x, y, z, u, v, w or $x, y, z, +1, u, v, w$ where x, y, z refer to the R orbital or lattice part of the $\{U||R\}$ spin point group operation and u, v, w refer to the spin part U. The integer ± 1 between both triplets in the second case is redundant and its value is $\det(U)$. The symmetry operations are converted into the matrix form $\{U||R\}$. In an mcif file the symmetry operations have the usual form of the symmetry operations of a magnetic point group, $x, y, z, \pm 1$. As in the previous case, x, y, z refer to the orbital part R and the U matrix can be obtained through the relation $U = \pm 1 \det(R)R$. The operations are converted to matrix form $\{U||R\}$. If the uploaded file is a scif file, in the next step the program reads the matrix that relates the bases in the orbital and spin spaces. If the uploaded file is an mcif file, by construction, both sets of matrices are expressed in the setting of the orbital space and the matrix is assumed to be the identity. Next the program checks whether the spin distribution is collinear, coplanar or non-coplanar, using the information in the file about the magnetic moments of the independent atoms in the asymmetric unit. First all the symmetry operations are applied to these independent moments to get the magnetic moments of all the atoms in the unit cell. If all spins are parallel or antiparallel the structure is collinear and the vector $\mathbf{n}$ is the direction parallel to the spins. If all the moments lie in the same plane the structure is coplanar and $\mathbf{n}$ is calculated through the cross product of the (non-parallel and not anti-parallel) moments of two atoms. Finally, the program checks that the Jahn symbol introduced by the user has the right format.

In Option B all the data are introduced manually by the user and the program checks first that these data have the right format: the Jahn symbol is correct, the M matrix that relates the bases $(\mathbf{a}, \mathbf{b}, \mathbf{c})$ and $(\mathbf{a}_s, \mathbf{b}_s, \mathbf{c}_s)$ is non-singular and at least one of the three components $\mathbf{n} = (n_1, n_2, n_3)$ of the direction parallel (perpendicular) to the distributions of spins in collinear (coplanar) structures is non-zero. The program accepts floating-point numbers as components of $\mathbf{n}$ but they are immediately transformed as a set of integer numbers that represent the same direction.

Every row in the box of generators must correspond to a pair of matrices $\{U||R\}$ that are non singular. Once the matrices have been constructed, it is checked that successive multiplications of the generators end in a finite number of symmetry operations so that the generated elements form a finite point group. In the present form of the program, whereas the point group of the operations R in the orbital space must be one of the 32 crystallographic point groups, the point group of the spin operations U can be not only one of the 32 crystallographic point groups, but also a finite point group that contains a 8-fold or a 12-fold proper or improper rotation (see Table S1 for a complete list of allowed non crystallographic point groups). Moreover, the program checks whether all the U operations keep invariant (or reverse) $\mathbf{n}$ in collinear or coplanar cases.

Once that the parameters of the input have been accepted, and the whole set of symmetry operations of the non trivial group have been determined, the U matrices, expressed initially in the $(\mathbf{a}_s, \mathbf{b}_s, \mathbf{c}_s)$

Table S1: List non-crystallographic point groups of the U operations allowed by the input of *STENSOR*.

8	$\overline{8}$	$8/m$	822	$8mm$	$\overline{8}m2$	$8/mmm$
12	$\overline{12}$	$12/m$	$12\,22$	$12mm$	$\overline{12}m2$	$12/mmm$

basis, are transformed into the setting of the orbital space through the given M matrix, $U' = MUM^{-1}$. If the user has not made use of this option, it is understood by the program that both R and U are expressed in the same basis and, then, the M matrix is the identity. In the collinear and coplanar cases the components of $\mathbf{n}$ are also transformed as $\mathbf{n'} = M\mathbf{n}$. Therefore, in the calculation of the tensor reduction, U' (from now on U) and R are expressed in the same reference system.

The calculation is divided in several steps, described in the following sections.

S1.1 Determination of the magnetic point group as a subgroup of the spin point group

As has been stressed in the main text, it is interesting to compare the form of a tensor under the given SpPG and under its subgroup whose operations form a maximal MPG. We have thus to identify those operations $\{U||R\}$ that satisfy $U = \pm R$ and that compose the MPG (Etxebarria *et al.*, 2025).

The operations of the point group can be expressed, in general, as the direct product of the nontrivial SpPG, $\mathrm{P_{NT}}$ and the spin-only group $\mathrm{P_{SO}}$,

$$\mathrm{P_S} = \mathrm{P_{NT}} \times \mathrm{P_{SO}}, \tag{1}$$

where $\mathrm{P_{SO}}$ contains all operations of the form $\{U||1\}$ and can, in turn, be expressed as the direct product of the *intrinsic* or trivial $\mathrm{P_{SOintr}}$ point group and $\mathrm{P_{SOG}}$

$$\mathrm{P_{SO}} = \mathrm{P_{SOG}} \times \mathrm{P_{SOintr}}. \tag{2}$$

The intrinsic group $\mathrm{P_{SOintr}}$ is the identity in non-coplanar distributions of spins, ${}^{m_{\mathbf{n}}}1$ in coplanar distributions, where $m_{\mathbf{n}}$ represents a mirror plane whose normal is parallel to $\mathbf{n}$ and ${}^{\infty_{\mathbf{n}}m}1$ in collinear distributions, where $\infty_{\mathbf{n}}$ is the rotation axis of order infinite parallel to $\mathbf{n}$. $\mathrm{P_{SOG}}$ contains, together with the identity, all the spin-only operations that do not belong to the trivial group. *STENSOR* splits the point group into the intrinsic part $\mathrm{P_{SOintr}}$ on one hand, and the rest of terms in the decomposition of $\mathrm{P_S}$. This group,$\mathrm{P_{NTE}}$, is the SpPG associated to the nontrivial SpSG, i.e., $\mathrm{P_{NTE}}$=$\mathrm{P_{NT}}\times\mathrm{P_{SOG}}$. Using this split of the point group the determination of the MPG is straightforward in the three cases of spin arrangements.

In collinear groups we take every operation of $\{U||R\}$ in $\mathrm{P_{NTE}}$ and check whether there exits some operation $\{U'||1\}$ in $\mathrm{P_{SOintr}}$:${}^{\infty_{\mathbf{n}}m}1$ such that $UU' = \pm R$. However, by construction, ${}^{\infty_{\mathbf{n}}m}1$

is the set of symmetry operations that keep $\mathbf{n}$ invariant, it is sufficient just to check whether the product $\pm U^{-1}R$ belongs to the intrinsic group, i.e., whether one of the following two conditions is fulfilled,

$$U^{-1}R\mathbf{n} = \theta\mathbf{n}, \tag{3}$$

with $\theta = \pm 1$. If the condition is fulfilled for θ, the symmetry operation $\{R, \theta \det(R)\}$ belongs to the MPG, being a unitary operation if $\theta \det(R) = 1$ and anti-unitary if $\theta \det(R) = -1$.

In coplanar systems the procedure is very similar. The operation $\{U||R\}$ belongs to the MSG if there exits some operation U' in the intrinsic point group (that contains only the identity and a mirror plane) such that $\theta U^{-1}R = U'$. This condition can be divided into two:

- $\theta U^{-1}R = 1$ with $\theta = \pm 1$.
- $\theta U^{-1}R = m_{\mathbf{n}}$ which is fulfilled if $(U^{-1}R)^2 = 1$, $\theta U^{-1}R \neq -1$, $\det(\theta U^{-1}R) = -1$ and $\theta U^{-1}R\mathbf{n} = -\mathbf{n}$.

In both cases the operation $\{R, \theta \det(R)\}$ belongs to the MPG.

In non-coplanar cases, as the intrinsic group is the identity, we have to select those operations $\{U||R\}$ of $\mathrm{P}_{\mathrm{NTE}}$ that fulfill the relation $U = \theta R$. The operation $\{R, \theta \det(R)\}$ belongs to the MPG.

Once all the operations of the MPG have been listed, the program identifies the magnetic point group. As the operations will not be expressed in general in the standard setting of the identified MPG, together with the symbol and number of the MPG, *STENSOR* provides a transformation matrix P from the setting where the original R operations are expressed to the standard setting of the MPG (Litvin, 2013) such that $R_s = P^{-1}RP$, being R_s the matrix of the symmetry operation in the standard setting.

S1.2 Tensor reduction under the magnetic point group

In this section we describe the algorithm used by *STENSOR* to calculate the constraints on a given tensor imposed by the magnetic point group. We can distinguish two steps in the tensor reduction. In the first step the (anti)symmetrization of the tensor is not considered, so only the point group operations are used and, in the second step, we add the conditions imposed by the (anti)symmetric subsets of components enclosed into [] and {} symbols.

S1.2.1 Tensor reduction by the operations of the MPG

If no (anti)symmetrization of indices is considered, one takes thus a tensor of rank r whose Jahn symbol (once the symmetrization and/or anti-symmetrization of some of its components have been removed) is $\mathrm{V}r$, $a\mathrm{V}r$, $e\mathrm{V}r$ or $ae\mathrm{V}r$.

Let $\{R^i, \theta^i\}$ be an element of the MPG with $\theta^i = 1, -1$ for unitary and anti-unitary operations,

respectively, and let $T_{i_1,i_2,\ldots,i_r}$ be the components of a tensor of rank r, with $i_j = 1, 2, 3$ for all $j = 1, \ldots, r$. Any element of the MPG introduces a set of restrictions on these components given by

$$T_{i_1,i_2,\ldots,i_r} = T_{i'_1,i'_2,\ldots,i'_r} = f^i R^i_{i'_1 i_1} R^i_{i'_2 i_2} \ldots R^s_{i'_r i_r} T_{i_1,i_2,\ldots,i_r}, \tag{4}$$

with $f^i = 1$, θ^i, $\det(R^i)$ or $\theta^i \det(R^i)$ for tensors of type Vr, aVr, eVr or aeVr, respectively. We now define a 3^r-dimensional vector $\mathbf{T}$ whose u component is $T_u = T_{i_1,i_2,\ldots,i_r}$ such that

$$u = 3^{r-1}(i_1 - 1) + 3^{r-2}(i_2 - 1) + \ldots + 3(i_{r-1} - 1) + (i_r - 1) + 1. \tag{5}$$

The vector $\mathbf{T}$ contains thus all the 3^r components of the tensor ordered in a specific way. It is straightforward to check that the relation (4) transforms into

$$T_u = T_{u'} = \mathcal{R}^i_{u'u} T_u, \tag{6}$$

being $\mathcal{R}^i$ the Kronecker (or direct) product of r matrices R^i times f^i,

$$\mathcal{R}^i = f^i \overbrace{R^i \otimes R^i \otimes \ldots \otimes R^i}^{r}. \tag{7}$$

It is possible to establish group isomorphisms between the point group operations R^i, the MPG operations $\{R^i|\theta^i\}$ and the $\mathcal{R}^i$ matrices such that,

$$\begin{array}{ccccl} R^1 & \rightarrow & \{R^1|\theta^1\} & \rightarrow & \mathcal{R}^1 = f^1 \overbrace{R^1 \otimes R^1 \otimes \ldots \otimes R^1}^{r}, \\ R^2 & \rightarrow & \{R^2|\theta^2\} & \rightarrow & \mathcal{R}^2 = f^2 \overbrace{R^2 \otimes R^2 \otimes \ldots \otimes R^2}^{r}. \end{array} \tag{8}$$

If

$$\{R^3|\theta^3\} = \{R^1|\theta^1\}\{R^2|\theta^2\} = \{R^3 = R^1R^2|\theta^3 = \theta^1\theta^2\}, \tag{9}$$

making use of the properties of the Kronecker product, it is immediate to check that,

$$\mathcal{R}^3 = \mathcal{R}^1\mathcal{R}^2. \tag{10}$$

It is important to note that equation (9) represents the product of symmetry operations without conjugation when $\theta^i = -1$. The consequences on the tensor reduction of the anti-unitary operations is carried out by the multiplication by θ^i in equation (4), when the Jahn symbol contains the a factor (Grimmer, 1993; Grimmer, 2017; Gallego *et al.*, 2019). Therefore, the $\mathcal{R}^i$ matrices of dimension $3^r \times 3^r$ form a 3^r dimensional representation ρ of the point group $\mathcal{P}$ formed by the R^i operations. The determination of the number of independent components of the vector $\mathbf{T} = (T_1, \ldots, T_{3^r})^T$ reduces thus to calculate the multiplicity of the identity representation in ρ, and the calculation of a set of basis vectors that transform under the unitary irreducible representation can be easily performed

making use of the projector operators (Bradley & Cracknell, 1972; Dresselhaus *et al.*, 2008). If we consider the identity irreducible representation, the projector reduces to,

$$(\mathbb{I}_{3^r})_{u'u} = \frac{1}{|\mathcal{P}|} \sum_{s=1}^{|\mathcal{P}|} \mathcal{R}^s_{u'u}, \tag{11}$$

where $\mathbb{I}_{3^r}$ is the $3^r \times 3^r$ identity matrix and $|\mathcal{P}|$ is the order of the point group $\mathcal{P}$. If one applies this projector into the vector that contains the tensor components the result is:

$$\mathbf{T} = \frac{1}{|\mathcal{P}|} \sum_{s=1}^{|\mathcal{P}|} \mathcal{R}^s \mathbf{T} \equiv \mathrm{P}_{\mathcal{P}} \mathbf{T}. \tag{12}$$

Thus, the number of independent components of the tensor is the rank $rank(\mathrm{P}_{\mathcal{P}})$ of the matrix

$$\mathrm{P}_{\mathcal{P}} = \frac{1}{|\mathcal{P}|} \sum_{s=1}^{|\mathcal{P}|} \mathcal{R}^s, \tag{13}$$

i.e., the number of linearly independent rows of $\mathrm{P}_{\mathcal{P}}$. Each row gives a relation between different components of the tensor although not all the rows are independent. If the tensor has no additional restrictions that come from the (anti)symmetry under the interchange of indices, this matrix has all the information about the reduced form of the tensor.

S1.2.2 Further tensor reduction under (anti)symmetrization of indices

Once the restrictions of the point group have been considered, in the second step the extra restrictions due to the (anti)symmetrization of indices are also taken into account.

Let the tensor be symmetric (or antisymmetric) under the interchange of the i_k and i_{k+1} components, i.e. the Jahn symbol of the symmetric tensor is $\mathrm{V}(k-1)[\mathrm{V}2]\mathrm{V}(r-k-1)$ (or $\mathrm{V}(k-1)\{\mathrm{V}2\}\mathrm{V}(r-k-1)$ in the antisymmetric case) where the parentheses have been added for clarity. In the 3^r dimensional space the r-rank tensor is described as $\mathbf{T} = (T_1, \ldots, T_{3^r})^T$ and the symmetric (antisymmetric) interchange of the components i_k and i_{k+1} is expressed through the following $\mathcal{S}$ ($\mathcal{A}$) matrices,

$$\begin{aligned} \mathcal{S} &= \overbrace{\mathbb{I}_3 \otimes \mathbb{I}_3 \otimes \ldots \otimes \mathbb{I}_3}^{k-1} \otimes S \otimes \overbrace{\mathbb{I}_3 \otimes \mathbb{I}_3 \otimes \ldots \otimes \mathbb{I}_3}^{r-k-1}, \\ \mathcal{A} &= \overbrace{\mathbb{I}_3 \otimes \mathbb{I}_3 \otimes \ldots \otimes \mathbb{I}_3}^{k-1} \otimes A \otimes \overbrace{\mathbb{I}_3 \otimes \mathbb{I}_3 \otimes \ldots \otimes \mathbb{I}_3}^{r-k-1}, \end{aligned} \tag{14}$$

with

$$S = \begin{pmatrix} 1 & 0 & 0 & 0 & 0 & 0 & 0 & 0 & 0 \\ 0 & 0 & 0 & 1 & 0 & 0 & 0 & 0 & 0 \\ 0 & 0 & 0 & 0 & 0 & 0 & 1 & 0 & 0 \\ 0 & 1 & 0 & 0 & 0 & 0 & 0 & 0 & 0 \\ 0 & 0 & 0 & 0 & 1 & 0 & 0 & 0 & 0 \\ 0 & 0 & 0 & 0 & 0 & 0 & 0 & 1 & 0 \\ 0 & 0 & 1 & 0 & 0 & 0 & 0 & 0 & 0 \\ 0 & 0 & 0 & 0 & 0 & 1 & 0 & 0 & 0 \\ 0 & 0 & 0 & 0 & 0 & 0 & 0 & 0 & 1 \end{pmatrix} \qquad A = \begin{pmatrix} -1 & 0 & 0 & 0 & 0 & 0 & 0 & 0 & 0 \\ 0 & 0 & 0 & -1 & 0 & 0 & 0 & 0 & 0 \\ 0 & 0 & 0 & 0 & 0 & 0 & -1 & 0 & 0 \\ 0 & -1 & 0 & 0 & 0 & 0 & 0 & 0 & 0 \\ 0 & 0 & 0 & 0 & -1 & 0 & 0 & 0 & 0 \\ 0 & 0 & 0 & 0 & 0 & 0 & 0 & -1 & 0 \\ 0 & 0 & -1 & 0 & 0 & 0 & 0 & 0 & 0 \\ 0 & 0 & 0 & 0 & 0 & -1 & 0 & 0 & 0 \\ 0 & 0 & 0 & 0 & 0 & 0 & 0 & 0 & -1 \end{pmatrix} \tag{15}$$

and $\mathbb{I}_3$ being the 3×3 identity matrix.

The two matrices $(\mathbb{I}_{3^r}, \mathcal{S})$ (or $(\mathbb{I}_{3^r}, \mathcal{A})$) form a group of order 2 so that, on the reduced form of the tensor components $(T_1, T_2, \ldots, T_{3^r})^T$ calculated in the first step and developed in section S1.2.1, one can apply the same arguments. First, it is possible do define the projectors,

$$\mathbb{I}_{3^r} = \frac{1}{2}\left(\mathbb{I}_{3^r} + \mathcal{S}\right) \qquad \text{and} \qquad \mathbb{I}_{3^r} = \frac{1}{2}\left(\mathbb{I}_{3^r} + \mathcal{A}\right) \tag{16}$$

in the symmetric and anti-symmetric case, respectively. The basis vectors can be calculated through the relations,

$$\mathbf{T} = \frac{1}{2}\left(\mathbb{I}_{3^r} + \mathcal{S}\right)\mathbf{T} \equiv \mathrm{P}_{\mathcal{S}}\mathbf{T} \qquad \text{and} \qquad \mathbf{T} = \frac{1}{2}\left(\mathbb{I}_{3^r} + \mathcal{A}\right)\mathbf{T} \equiv \mathrm{P}_{\mathcal{A}}\mathbf{T}. \tag{17}$$

Using the relation (12) the symmetry reduction due to the MPG and the intrinsic symmetry of the tensor can be written as,

$$\mathbf{T} = \mathrm{P}_{\mathcal{P}}\mathrm{P}_{\mathcal{S}}\mathbf{T} \qquad \equiv \mathrm{P}_{\mathcal{PS}}\mathbf{T} \qquad \text{and} \qquad \mathbf{T} = \mathrm{P}_{\mathcal{P}}\mathrm{P}_{\mathcal{A}}\mathbf{T} \equiv \mathrm{P}_{\mathcal{PA}}\mathbf{T} \tag{18}$$

in the symmetric and anti-symmetric case, respectively. The matrices $\mathrm{P}_{\mathcal{PS}}$ and $\mathrm{P}_{\mathcal{PA}}$ contain thus all the information about the final form of the (symmetric in the first case and anti-symmetric in the second case) tensor: $rank(\mathrm{P}_{\mathcal{PS}})$ and $rank(\mathrm{P}_{\mathcal{PA}})$ give the number of independent components and each non-zero row of the matrices gives a set of constraints between the non-zero components of the tensor.

If the Jahn symbol contains more that one pair of symmetric and/or anti-symmetric components, for instance [V2][V2], [V2]V2{V2}, {V2}V{V2}, etc..., for every pair it is possible to construct the $\mathcal{S}$ or $\mathcal{A}$ matrices and, considering the groups of index 2 formed by every such matrices and the identity, to calculate the further symmetry restrictions using exactly the same algorithm. Therefore, the final set of independent tensor components and the relations between the non-zero components

can be determined by a single final matrix,

$$\mathrm{P}_{\mathcal{P}\mathcal{S}_1\ldots\mathcal{S}_k\mathcal{A}_1\ldots\mathcal{A}_\ell} = \mathrm{P}_{\mathcal{P}}\mathrm{P}_{\mathcal{S}_1}\ldots\mathrm{P}_{\mathcal{S}_k}\mathrm{P}_{\mathcal{A}_1}\ldots\mathrm{P}_{\mathcal{A}_\ell}, \tag{19}$$

where k is the number of symmetric pairs of indices and ℓ is the number of anti-symmetric pairs.

The $\mathcal{S}$ matrix defined by equations (14) and (15) represents the interchange of two components of a tensor. However, there exist physical properties whose tensor is invariant under the permutation of 3 of more components. For instance, the *optical rectification* in non-dissipative media without dispersion is described by a 3-rank tensor $\chi(0;\omega,-\omega)_{ijk}$ symmetric under the permutation of the three components (Jahn symbol [V3]) or the *electric-field induced second-harmonic generation* in non-dissipative media without dispersion is described by a 4-rank tensor $\chi(2\omega;0,\omega,\omega)_{ijk\ell}$ symmetric under the permutation of the 4 indices (Jahn symbol [V4]). In general, one can consider a Jahn symbol that contains as part of the whole symbol [Vn] with $n > 2$, being $n!$ the number of possible permutations of indices. These permutations form a group isomorphic to the group of permutations of grade n. Let's consider that the Jahn symbol of such a tensor is Vp[Vn]V$(r-p-n)$, with $p \geq 0$, $n > 2$ integer numbers and r the rank of the tensor. The $n!$ permutations can be obtained by a set of $(n-1)$ generators: the operations that represent the interchange of the components $(p+1, p+2)$, $(p+2, p+3)$,... and $(p+n-1, p+n)$, constructed as shown by equations (14) and (15). The successive multiplication of these $(n-1)$ matrices by each other gives as a result the $n!$ matrices that represent the different permutations. As these matrices form a finite group, it is possible to construct a projector equivalent to the projector of equation (11), that finally defines an extra matrix $\mathrm{P}_{\mathcal{S}n}$. This matrix can be added to the final list of matrices (19) as an extra multiplicative factor.

Finally, it is necessary to consider those tensors whose intrinsic symmetry contains the invariance under the permutation of pairs of indices. For instance, the *elastic compliance* and the *elastic stiffness* tensors of rank 4 have as Jahn symbol [[V2][V2]]. These tensors are symmetric under the interchange of the first two indices, under the interchange of the last two indices (these symmetries have been considered before and they contribute to the final matrix (19) with two factors) and also under the interchange of the two pairs of indices. This operation can also be represented by a matrix of the form given by equation (14), but instead of the matrix $\mathcal{S}$ of dimension 9×9 given in equation (15), a 81×81 matrix will substitute the central $E \otimes S \otimes E$ subset in equation (14). The construction of this matrix is straightforward and, together with the identity matrix, it forms a group of index 2. Following the same procedure as with the other symmetries, it will add an extra (multiplicative) term in the final matrix (19).

S1.2.3 Some considerations on the algorithm

To finish this section several considerations about the used algorithm should be added.

- The procedure outlined in the previous two sections allows one to calculate the symmetry reduction of any tensor that may contain intrinsic symmetries or not under any MPG. The problem reduces to the calculation of the $3^r \times 3^r$ matrix of equation (19). Every non-zero row of this matrix contains a relation between the tensor coefficients T_u with $u = 1, \ldots, 3^r$ ordered according to the 1:1 map given by equation (5). However, in general, these non-zero rows can involve intricate relations between the coefficients. To simplify the final expression of the tensor, *STENSOR* performs the *row reduction* (or Gauss decomposition) to convert the matrix (19) into its *reduced row echelon form* whose main properties are: it is an upper triangular matrix, only the first $r_P = rank(\mathrm{P}_{P\mathcal{S}_1 \ldots \mathcal{S}_k \mathcal{A}_1 \ldots \mathcal{A}_\ell})$ rows have at least one nonzero component, the first nonzero element in these non-zero rows is 1 and this is the only nonzero component in its column.

  Using the final echelon form of the matrix (19), the table of components of the tensor can be easily constructed. Each non-zero row corresponds to an independent component of the final tensor. If we denote as $\mathcal{P}^e$ the $r_P \times 3^r$-dimensional matrix that contains the non-zero rows of the echelon matrix obtained by Gauss decomposition of the matrix (19), the tensor components are,

$$(T_1, T_2, \ldots, T_{3^r}) = (a_1, a_2, \ldots, a_{r_P})\, \mathcal{P}^e, \tag{20}$$

  where a_i are the r_P independent parameters. To assign the label to each independent coefficient, we identify the position u (the column index) of the first non-zero coefficient of the row that, by construction, takes the value 1, and identify though equation (5) the values of the indices $i_1, \ldots, i_r$. The label of the corresponding independent coefficient in *STENSOR* is

$$a_j = c^j_{i_1,\ldots,i_r} \qquad j = 1, \ldots, r_P. \tag{21}$$

- When the MPG belongs to the trigonal or hexagonal crystal system, usually the symmetry operations are introduced in the standard hexagonal setting, with $a = b$, $\alpha = \beta = 90°$ and $\gamma = 120°$. As the final form of the tensor components are given in an orthogonal setting, the symmetry operations and the direction $\mathbf{n}$ in collinear and coplanar cases are previously transformed into an orthogonal setting with $a' = b'$ and $\alpha' = \beta' = \gamma' = 90°$. In principle, there are 6 possible symmetry equivalent orientations of the final setting with respect to the original hexagonal setting. In most cases, the final form of the non-zero but dependent tensor coefficients can be expressed as linear combinations of the independent coefficients using rational coefficients. However, after a rotation of the symmetry axes by an angle multiple of $2\pi/6$, although the new directions are equivalent to the original ones, the mentioned linear combinations will include non-fractional coefficients. In the trigonal and hexagonal groups *STENSOR* checks the 6 symmetry equivalent pairs of orthogonal axes perpendicular to the 3-fold axis to choose the basis in which the final tensor takes the simplest form.

- The memory and cpu-time requirements in the calculation depends strongly on the rank of

the given tensor. For rank-8 tensors, for instance, the matrices involved in the calculation given by equations (7) or (14) are of dimension 6561×6561. If the order of the MPG is high, the full calculation can take a long time, and the program will stop if it does not reach the final result in a predefined time limit.

However, the memory and time requirements can be strongly reduced using *sparse arrays*, implemented in most program codes of linear algebra. It is particularly useful when the matrices involved in the calculations have a relatively large number of null components. Using sparse arrays, a matrix of $n \times n$ components is described by an array that contains only the non-zero components and thus, operations as sums or multiplications of matrices are most effectively performed using these arrays, avoiding a large number of multiplications by 0. The use of sparse arrays is particularly useful when the matrices involved represent crystallographic symmetry operations. Except in trigonal and hexagonal point groups, when described in the standard setting (or in most reasonable settings), only 3 components of the R matrices out of 9 (1/3 factor) are different from 0. In the Kronecker product of r such matrices (equations (7) and (14)), the proportion of non-zero coefficients is $(1/3)^r$, i.e., only 0.01% of coefficients for $r = 8$, for instance. The matrices of the point groups in the trigonal and hexagonal crystal systems expressed in an orthogonal reference system have 3 or 5 non-zero components. The use of sparse arrays is not so advantageous in these crystal systems but still very significant. *STENSOR* uses sparse arrays in all the calculations with matrices.

- Except when the rank of the tensor is 2, *STENSOR* uses the Voigt form when the Jahn symbol contains one or several symmetric terms of type [V2]. In those cases, it uses the usual assignation $11 \to 1$, $22 \to 2$, $33 \to 3$, $23, 32 \to 4$, $13, 31 \to 5$ and $12, 21 \to 6$ to reduce the number of dependent components. It also uses the Voigt form of as many pairs of indices as possible for tensors that contain terms of the type [Vn] with $n > 3$. The assignation is direct, i.e., $c_{ij} \to c_\alpha$ with $i, j = 1, 2, 3$ and $\alpha = 1, \ldots, 6$ with no assignation of factors 1/2 or 1/4 to specific tensors as it is often the case (Nye, 1985).

S1.3 Tensor reduction under the spin point group

The calculation of the tensor reduction under the whole spin group can be divided into three steps, being the first two almost identical to the ones followed in the previous section to calculate the reduction under the MPG. First, the constraints imposed by $\mathrm{P_{NTE}}$ are expressed through a matrix equivalent to equation (12), next the constraints due to the (anti)symmetrization under the interchange of sets of indices are added as explained in section (S1.2.2) and finally, as a third and final step, the additional reduction imposed by the trivial point group in collinear and coplanar spin arrangements is also added.

S1.3.1 Tensor reduction by the operations of the SpPG

The reduction of the components of a tensor under the SpPG is carried out following the procedure explained in section (S1.2.1), but some expressions must be slightly modified. The constraints imposed by an operation $\{U||R\}$ of the SpPG on a rank-1 tensor depends on the type on tensor. In Etxebarria *et al.* (2025) four different types of ferroic tensors of rank-1 tensors were described: V (polar vector, odd under inversion and even under time-reversal), eV (axial vector, even under both inversion and time-reversal), M (axial magnetic vector, even under inversion and odd under time-reversal) and T (toroidic moment, odd under both inversion and time-reversal). In general, the components of a tensor of rank r transform as the product of rank-1 tensors, where each component can be of V- or M-type. In this section we are not considering tensors that include components that transform as T (these tensors will be analyzed in section (S1.3.3)).

As in section (S1.2.1), in this first step the (anti)symmetrization of the tensor under the interchange of indices is not considered and therefore, in general, the Jahn symbol to be considered is J, eJ, aJ or aeJ, where J represents a set of V and M components. The procedure developed in this section is exactly the same for the four types of tensors. The V components transform under R and the M components transform under U when the symmetry operation of the point group $\{U||R\}$ is considered. The symmetry constraints imposed by the operation $\{U^i||R^i\}$ is thus,

$$T_{i_1,i_2,\ldots,i_r} = T_{i'_1,i'_2,\ldots,i'_r} = f^i (U|R)^i_{i'_1 i_1} (U|R)^i_{i'_2 i_2} \ldots (U|R)^i_{i'_r i_r} T_{i_1,i_2,\ldots,i_r}, \tag{22}$$

where $(U|R)^i_{i'_j i_j} = R_{i'_j i_j}$ or $(U|R)^i_{i'_j i_j} = U_{i'_j i_j}$ if the j-th term in the sequence of letters in the Jahn symbol is V or M, respectively. The expression (7) must be generalized to,

$$\mathcal{R}^i = f^i \overbrace{(U|R)^i \otimes (U|R)^i \otimes \ldots \otimes (U|R)^i}^{r}. \tag{23}$$

Note that, in principle, the factor f^i is different in equations (7) and (23). The sum of the matrices (23) for all the symmetry operations of the SpPG (equation (13)) contains all the information about the symmetry reduction of the tensor due to the SpPG. In general, the order of the SpPG in equation (23) is higher that the number of operations of the MPG in equation (12).

The (anti)symmetrization of the tensor in the context of MPG and SpPG is exactly the same: the substitution M→ aeV does not change the (anti)symmetric character of the tensor under the interchange of indices: both [Vn] and [Mn] transform into [Vn] (in the second case accompanied by ae if n is odd) and both {V2} and {M2} transform into {V2}. Therefore, all the factors in the final matrix given by equation (19) are exactly the same, except the first one, as explained in this section.

S1.3.2 Tensor reduction by the operations of the trivial group

To obtain the final form of the tensor, the additional restrictions imposed by the trivial group must also be considered. In non-coplanar groups the trivial group is the identity and it does not introduce new restrictions. The matrix (19) contains all the information about the tensor, which takes the form given by equations (20) and (21).

The restrictions added by the coplanar or collinear groups can be analyzed as follows.

- In coplanar groups, apart from the identity, the only operation of the trivial group is a mirror plane perpendicular to the direction $\mathbf{n} = (n_1, n_2, n_3)$. As stated above, if the input parameters are not given in an orthogonal basis (usually it is the case in trigonal and hexagonal crystal systems) they are transformed into an orthogonal one, so at this point (n_1, n_2, n_3) are the components of the direction in an orthogonal basis. The matrix that represents a mirror plane perpendicular to $\mathbf{n}$ is,

$$m_{\perp\mathbf{n}} = \frac{1}{n^2}\begin{pmatrix} n^2 - 2n_1^2 & -2n_1n_2 & -2n_1n_3 \\ -2n_1n_2 & n^2 - 2n_2^2 & -2n_2n_3 \\ -2n_1n_3 & -2n_2n_3 & n^2 - 2n_3^2 \end{pmatrix}. \qquad (24)$$

  It is easy to check that the matrix (24) represents the mirror plane because $m_{\perp\mathbf{n}}^2 = m_{\perp\mathbf{n}}.m_{\perp\mathbf{n}}^T = 1$, $\det(m_{\perp\mathbf{n}}) = -1$ and $m_{\perp\mathbf{n}}\mathbf{n} = -\mathbf{n}$. To determine the restrictions imposed by the trivial group one can follow the procedure explained in section (S1.2.1). For this operation, in the Kronecker product (23) all the terms of the product are $(U|R) \to \mathbb{I}_3$ or $(U|R) \to U = m_{\mathbf{n}}$ in equation (24), depending on the corresponding letter V or M, respectively, in the Jahn symbol. Together with the identity, the resulting matrix $\mathcal{R}_{m_{\perp\mathbf{n}}}$ forms a group of order 2 and the corresponding projector (11) can be defined. Finally, the symmetry restrictions of the trivial group are included in the matrix,

$$P_{m_{\perp\mathbf{n}}} = \frac{1}{2}\left(\mathbb{I}_{3^r} + \mathcal{R}_{m_{\perp\mathbf{n}}}\right), \qquad (25)$$

  which can be added as a multiplicative factor to the matrix (19) that contains all the symmetry restrictions considered so far. After the row reduction of the final matrix, the final the form of the output is given by equations (20) and (21).

- In the collinear case the trivial group contains a rotation axis of order infinite and a set of infinite parallel planes that contain the axis. The trivial group can be expressed as the direct product of two subgroups: the subgroup that contains all the operations that represent rotations around the unique axis (of arbitrary angle) and a subgroup of order 2 that contains the identity and a mirror plane

$$\infty_{\mathbf{n}} m = (E + m_{\parallel\mathbf{n}}) \otimes \infty_{\mathbf{n}}, \qquad (26)$$

where $m_{\parallel\mathbf{n}}$ is any mirror plane that satisfies $m_{\parallel\mathbf{n}}\mathbf{n} = \mathbf{n}$. The symmetry reduction of the tensor due to the trivial group has thus two contributions that can be analyzed separately: on the one hand the restrictions under the subgroup formed by the identity and a mirror plane and on the other hand the restrictions imposed by the rotations $\infty_{\mathbf{n}}$.

The algorithm to calculate the restrictions due to the mirror plane is exactly the same as the one used in the coplanar case. One matrix that represents a mirror plane that keeps invariant the direction (n_1, n_2, n_3) is,

$$m_{\parallel\mathbf{n}} = \begin{cases} \frac{1}{n_1^2+n_2^2}\begin{pmatrix} n_1^2 - n_2^2 & 2n_1n_2 & 0 \\ 2n_1n_2 & n_2^2 - n_1^2 & 0 \\ 0 & 0 & n_1^2 + n_2^2 \end{pmatrix} & \text{if } n_1^2 + n_2^2 \neq 0 \\ \begin{pmatrix} -1 & 0 & 0 \\ 0 & 1 & 0 \\ 0 & 0 & 1 \end{pmatrix} & \text{if } n_1^2 + n_2^2 = 0 \end{cases} . \tag{27}$$

After the calculation of the $\mathcal{R}_{m_{\parallel\mathbf{n}}}$ matrix as the Kronecker product (23) with $(U|R) \to \mathbb{I}_3$ or $(U|R) \to U = m_{\parallel\mathbf{n}}$, depending on the corresponding letter V or M, respectively, in the Jahn symbol, the matrix

$$P_{m_{\parallel\mathbf{n}}} = \frac{1}{2}\left(\mathbb{I}_{3^r} + \mathcal{R}_{m_{\parallel\mathbf{n}}}\right) \tag{28}$$

is added as a multiplicative factor to the matrix (19).

To determine the restrictions imposed by the second group in the direct product (26) the strategy used by *STENSOR* is different from the one followed with all other symmetries in sections (S1.2) and (S1.3). The order of the group $\infty_{\mathbf{n}}$ is infinite and it is not possible to define a projector as in equation (11). For this group, *STENSOR* calculates first the final form of the tensor due to the rest of symmetry operations: the operations of $\mathrm{P}_{\mathrm{NTE}}$, the (anti)symmetric sets (if any) and the mirror plane (27). The partially reduced form of the tensor takes the form of equations (20) and (21). Then, on this final form, *STENSOR* establishes the set of linear equations (6) for the $\mathcal{R}$ matrix of one generator (arbitrary rotation of angle φ). In general, these linear equations will introduce relations between the so far independent $c^j_{i_1,\ldots,i_r}$ parameters. Finally *STENSOR* reconstructs the output taking into consideration these relations.

The matrix that represents a rotation of angle φ around an axis parallel to the direction (n_1, n_2, n_3) is,

$$C_\varphi = \frac{1}{n^2}\begin{pmatrix} n_1^2 + \left(n_2^2 + n_3^2\right)\cos\varphi & n_1n_2\left(1-\cos\varphi\right) - nn_3\sin\varphi & n_1n_3\left(1-\cos\varphi\right) + nn_2\sin\varphi \\ n_1n_2\left(1-\cos\varphi\right) + nn_3\sin\varphi & n_2^2 + \left(n_1^2 + n_3^2\right)\cos\varphi & n_2n_3\left(1-\cos\varphi\right) - nn_1\sin\varphi \\ n_1n_3\left(1-\cos\varphi\right) - nn_2\sin\varphi & n_2n_3\left(1-\cos\varphi\right) + nn_1\sin\varphi & n_3^2 + \left(n_1^2 + n_2^2\right)\cos\varphi \end{pmatrix}. \tag{29}$$

It can be checked that it is the right matrix: $C_\varphi . C_\varphi^T = 1$, $\det(C_\varphi) = 1$, $C_\varphi . C_\varphi = C_{2\varphi}$ and $C_\varphi \mathbf{n} = \mathbf{n}$.

As a generator of the group, it can be considered an infinitesimal angle $\varphi \ll 1$ and perform a series expansion of the matrix (29) keeping only up to the linear term. The matrix is

$$C_\varphi \simeq \mathbb{I}_3 + \varphi\omega, \tag{30}$$

with

$$\omega = \frac{1}{n}\begin{pmatrix} 0 & -n_3 & n_2 \\ n_3 & 0 & -n_1 \\ -n_2 & n_1 & 0 \end{pmatrix}. \tag{31}$$

The Kronecker product (23) up to the linear term takes the form

$$\mathcal{R}^\infty = \mathbb{I}_{3^r} + \varphi\Omega, \tag{32}$$

being Ω a $3^r \times 3^r$-dimensional numerical (independent of φ) matrix. Using vector notation, the linear set of equations (6) takes de form,

$$\mathbf{T} = (\mathbb{I}_{3^r} + \varphi\Omega)\,\mathbf{T} \qquad \rightarrow \qquad \Omega\mathbf{T} = 0. \tag{33}$$

Taking the last form (20) of $\mathbf{T}$, the set of homogeneous linear equations (33) can be written as,

$$(a_1, a_2, \ldots, a_r)\,\mathcal{P}^e\Omega^T = 0. \tag{34}$$

In general, this set of equations will introduce linear relations between the *independent* coefficients a_i. Using these relations between the coefficients, the final form of the tensor is,

$$(T_1, T_2, \ldots, T_{3^r}) = \left(a'_1, a'_2, \ldots, a'_{r'_P}\right)\mathcal{P}'^e, \tag{35}$$

with $r'_P \leq r_P$ and being $\mathcal{P}'^e$ an echelon matrix. As in equations (20) and (21), the label of the a'_j coefficients shown by *STENSOR* is $a'_j = c^j_{i_1,\ldots,i_r}$, being $i_1, \ldots, i_r$ the set of indices that correspond to the first non-zero value of the jth row of $\mathcal{P}'^e$.

S1.3.3 Symmetry constraints of tensors that contain toroidic components

The moment of magnetization or *toroidic* moment $\mathbf{T} = \mathbf{r} \times \mathbf{M}$ is odd both under spatial inversion and under time-reversal symmetry. The transformation properties of these quantities involve both R space and U spin operations and can be obtained as the antisymmetric part of the magnetoelectric tensor (direct or inverse effect) (Spaldin *et al.*, 2008). Following Etxebarria *et al.* (2025) and taking the inverse magnetoelectric effect as the reference tensor, whose Jahn symbol is MV, we denote the Jahn symbol of the toroidic moment as {MV}, reflecting the fact that it transforms as the antisymmetric part of the magnetoelectric tensor α^T_{ij}. *STENSOR* performs the symmetry reduction of a tensor that contains, as a part of its Jahn symbol, one of several components that transform as the toroidic moment, in two steps:

- First, it determines the symmetry reduction of the tensor T' with the Jahn symbol that results after the substitution {MV}→MV, following the procedure described in section S1.2 for the MPG and section S1.3 for the SpPG. The output contains the corresponding two tables.
- Taking the general form for the SpPG (second table in the previous step), *STENSOR* makes the antisymmetric reduction using the Levi-Civita tensor (Etxebarria *et al.*, 2025),

$$T_{i_1,\dots,i_{j-1},k,i_{j+2},\dots,i_r} = \frac{1}{2}\varepsilon_{k,i_j,i_{j+1}} T'_{i_1,\dots,i_{j-1},i_j,i_{j+1},i_{j+2},\dots,i_r}, \tag{36}$$

  where it has been assumed that the M and V symbols inside {MV} occupy the positions i_j and i_{j+1} in the Jahn symbol. If the Jahn symbol contains more than one {MV} term, it must be performed a reduction of the type given by equation (36) for each term.

At the end of the output, a third table shows the general form of the quantity that contains toroidic terms.

S2 Example: symmetric spin contribution to the Hall effect in $NiCr_2O_4$

This section includes a detailed application of the algorithm explained in section S1 using an example. The physical property chosen is the symmetric part of the Hall effect (or linear magnetoresistence) R^s_{ijk}, whose tensor has Jahn symbol [V2]M and the compound is $NiCr_2O_4$ (Tomiyasu & Kagomiya, 2004) (entry 0.4 in *MAGNDATA*). The identified spin space group (Chen *et al.*, 2024) is collinear with symbol $P\,^14_1/^1a\,^1m\,^1d\,^{\infty_{110}m}1$ (N. 141.141.1.1). The non-trivial group $\mathrm{P_{NT}}$ is $^14/^1m\,^1m\,^1m$ and the spin-only group is $^{\infty_{110}m}1$ with the spins aligned along the $[1,1,0]$ direction.

Fig. 2 in the main text shows the input page of *STENSOR* where the data of the example have been introduced. The red text and the arrows have been added to the figure to show the data introduced by the user. As it can be seen in the figure, the following three operations have been introduced as generators of $\mathrm{P_{NT}}$

$$\begin{array}{ll} -y,x,z,+1,u,v,w & \{1||4^+_{001}\} \\ x,y,-z,+1,u,v,w & \{1||m_{001}\} \\ -x,y,z,+1,u,v,w & \{1||m_{100}\} \end{array} \tag{37}$$

It has been assumed that the basis vectors in the orbital and spin spaces are exactly the same. If the U and R matrices of the spin and orbital spaces, respectively, were expressed in different bases, clicking on the button *spin basis* a 3×3 table would emerge. In that table the user can introduce the components of the matrix that relates both reference systems. By default the program assumes that this matrix is the identity.

S2.1 Determination of the MPG

The complete set of operations of $\mathrm{P_{NT}}$ calculated by *STENSOR* are,

$$\begin{array}{llllllll} \{1||1\} & \{1||4^{+}_{001}\} & \{1||2_{001}\} & \{1||4^{-}_{001}\} & \{1||\bar{1}\} & \{1||\bar{4}^{+}_{001}\} & \{1||m_{001}\} & \{1||\bar{4}^{-}_{001}\} \\ \{1||m_{100}\} & \{1||m_{1\bar{1}0}\} & \{1||m_{010}\} & \{1||m_{110}\} & \{1||2_{100}\} & \{1||2_{1\bar{1}0}\} & \{1||2_{010}\} & \{1||2_{110}\} \end{array} \tag{38}$$

Note that the complete set of operations are the direct product of the operations in the list (38) and the operations $\{U||1\}$ of the trivial group.

Following the procedure developed in section (S1) the program selects from the list (38) those that fulfill the condition (3) with $\mathbf{n} = (1, 1, 0)$ in this particular example. As the spin operation U is the identity in all the cases, the condition transforms into $R\mathbf{n} = \theta\mathbf{n}$ with $\theta = \pm 1$ for all the operations. Only the following 8 operations fulfill one of the conditions,

$$\begin{array}{l|l|l|l} 1\mathbf{n} = \mathbf{n} \to \{1, 1\} & 2_{001}\mathbf{n} = -\mathbf{n} \to \{2_{001}, -1\} & \bar{1}\mathbf{n} = -\mathbf{n} \to \{\bar{1}, 1\} & m_{001}\mathbf{n} = \mathbf{n} \to \{m_{001}, -1\} \\ m_{1\bar{1}0}\mathbf{n} = \mathbf{n} \to \{m_{1\bar{1}0}, -1\} & m_{110}\mathbf{n} = -\mathbf{n} \to \{m_{110}, 1\} & \{2_{1\bar{1}0}, -1\}\mathbf{n} = -\mathbf{n} \to \{2_{1\bar{1}0}, -1\} & 2_{110}\mathbf{n} = \mathbf{n} \to \{2_{110}, 1\} \end{array} \tag{39}$$

On the right side of each $R\mathbf{n} = \theta\mathbf{n}$ condition it has been shown the resulting operation of the MPG in the usual notation $\{R, \theta\}$. The 8 operations form the MPG $mm'm'$ (N. 8.4.27), but they are not expressed in the standard setting of this MPG. The two planes parallel to the z axis are rotated 45° with respect to the planes in the standard description. Together with the identification of the symbol and sequential number of the MPG, *STENSOR* gives also a transformation matrix to the standard setting. Fig. (S1) shows the first part of the output given by *STENSOR*.

The first part of the output includes the input parameters, the identification of the crystal system, information about the setting used for the orbital part of the symmetry operations and a link to the complete set of symmetry operations of $\mathrm{P_{NT}}$ (list of operations in equation (38) in the $x, y, z, +1, u, v, w$ format). Just below the information related with the input data, the program shows the identified MPG together with the transformation matrix P that relates the operations in (39) with the operations of the standard setting of $mm'm'$, i.e., $\{R_s, \theta\} = \{P^{-1}RP, \theta\}$, where R is the rotational part of the operations in equation (39) and R_s is the corresponding operation in the standard description. The output also shows the Jahn symbol in the context of MPG of the tensor given. In this example, doing the substitution M→ aeV, the Jahn symbol to be used to calculate the symmetry reduction by the MPG is ae[V2]V.

S2.2 Tensor reduction by the MPG

Next, the program performs the tensor reduction by the MPG that, as explained in sections (S1.2.1) and (S1.2.2), consists in the determination of the matrices $\mathrm{P}_{\mathcal{P}}$ in equation (13) and $\mathrm{P}_{\mathcal{S}}$ in equation (17), which reproduce the symmetry reduction due to the point group and the intrinsic symmetry of the tensor, respectively. To calculate the first one, the program first does the Kronecker products (7) of the 8 operations of the MPG in equation (39) with $r = 3$ and $f^i = \theta_i \det(R^i)$ for the tensor chosen ae[V2]V. The matrix (13) is just the sum of these 8 matrices. The matrix $\mathcal{S}$ that represents

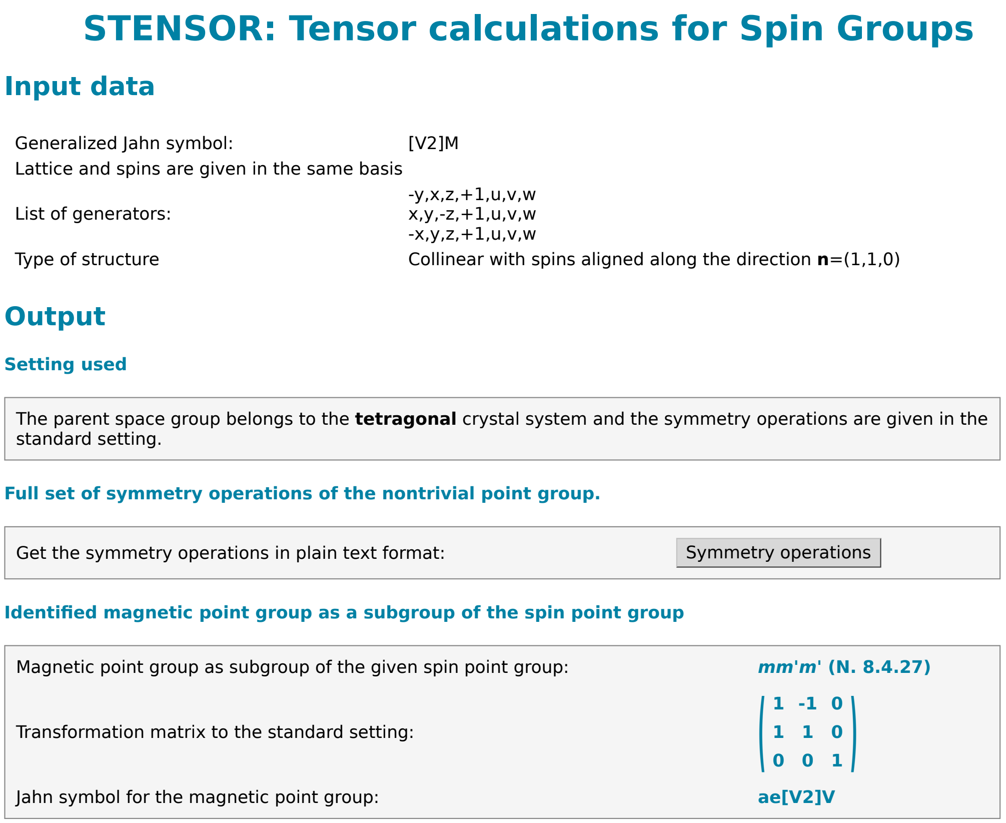

Figure S1: First part of *STENSOR*'s output for the input given in Fig. (2). It shows the input data, a link to the complete list of symmetry operations of P_{NT} in plain text, information about the crystal system, the identified MPG, the transformation matrix from the setting given by the user to the standard setting of the MPG and the Jahn symbol for the MPG.

the interchange of the first two indices of the tensor (equations (14) and (15)) is in this case $=S\otimes\mathbb{I}$. This allows to construct the matrix $P_{\mathcal{S}}$ in equation(17). The matrix that allows to determine the full reduction of the ae[V2]V for the MPG of equation (39) is the product of both matrices (equation (17)). After the row reduction of this product, and keeping only the non-zero rows, the final matrix has only 5 rows, shown below the line in table (S2). The table also lists the 27 coefficients of the tensor above the line, following the convention given by equation (5).

Using the Voigt correspondence for the two first indices, reordering the columns to align the newly defined components, and performing again the row reduction of the matrix, the final result is shown in table (S3). Every row corresponds to an independent component of the tensor. On the left of the table, it has been added another column with the label assigned to each independent coefficient. The natural election of the label is the tensor coefficient that corresponds to the first non-zero value in the row (it is always 1 by construction of the echelon form of the matrix). The final value of every element of the tensor is the sum of matrix elements of its column, multiplied by the corresponding coefficient. Fig. (S1) shows the second part of the output given by *STENSOR* and displays the final form of the tensor (table (S3)) under the reduction of the MPG and the intrinsic symmetry,

Table S2: Above the line, the components of a rank-3 tensor are indicated (the indices are aligned vertically to save horizontal space), in the order given by expression (5). Below the line, the table contains the non-zero components of the matrix that gives the symmetry reduction of the tensor with Jahn symbol ae[V2]V under the MPG with elements in equation (38) (after the row reduction).

c	c	c	c	c	c	c	c	c	c	c	c	c	c	c	c	c	c	c	c	c	c	c	c	c	c	c
1	1	1	1	1	1	1	1	1	2	2	2	2	2	2	2	2	2	3	3	3	3	3	3	3	3	3
1	1	1	2	2	2	3	3	3	1	1	1	2	2	2	3	3	3	1	1	1	2	2	2	3	3	3
1	2	3	1	2	3	1	2	3	1	2	3	1	2	3	1	2	3	1	2	3	1	2	3	1	2	3
1	0	0	0	0	0	0	0	0	0	0	0	0	1	0	0	0	0	0	0	0	0	0	0	0	0	0
0	1	0	0	0	0	0	0	0	0	0	0	1	0	0	0	0	0	0	0	0	0	0	0	0	0	0
0	0	0	1	1	0	0	0	0	1	1	0	0	0	0	0	0	0	0	0	0	0	0	0	0	0	0
0	0	0	0	0	0	0	0	1	0	0	0	0	0	0	0	0	1	0	0	1	0	0	1	0	0	0
0	0	0	0	0	0	0	0	0	0	0	0	0	0	0	0	0	0	0	0	0	0	0	0	1	1	0

Table S3: Reduced form of table S2, using Voigt-like notation. The columns have been reordered to keep the 1,2,... sequence of indices and the row reduction has been applied again. On the left, a label has been assigned to each independent row.

	c	c	c	c	c	c	c	c	c	c	c	c	c	c	c	c	c	c
	1	1	1	2	2	2	3	3	3	4	4	4	5	5	5	6	6	6
	1	2	3	1	2	3	1	2	3	1	2	3	1	2	3	1	2	3
c_{11}	1	0	0	0	1	0	0	0	0	0	0	0	0	0	0	0	0	0
c_{12}	0	1	0	1	0	0	0	0	0	0	0	0	0	0	0	0	0	0
c_{31}	0	0	0	0	0	0	1	1	0	0	0	0	0	0	0	0	0	0
c_{43}	0	0	0	0	0	0	0	0	0	0	0	1	0	0	1	0	0	0
c_{61}	0	0	0	0	0	0	0	0	0	0	0	0	0	0	0	1	1	0

expressed in the Voigt-like form.

S2.3 Tensor reduction by the SpPG

Following the method described in section (S1.3), *STENSOR* calculates three matrices:

- The matrix (13) which contains the information about the symmetry restrictions imposed by $\mathrm{P_{NT}}$. The matrix $\mathcal{R}^s$ for the tensor chosen [V2]M is,

$$\mathcal{R}^s = R^s \otimes R^s \otimes U^s \qquad (40)$$

  and the summation must be calculated for all the 16 members of the SpPG (38).

- The matrix $\mathrm{P}_{\mathcal{S}}$ in equation (17) which contains the information about the restrictions imposed by the intrinsic symmetry. This matrix is exactly the same as the matrix used in section S2.2 to calculate the restrictions under the MPG.

- The matrix $P_{m_{\|\mathbf{n}}}$ in equation (28) that gives the restrictions of a single mirror plane of the trivial group in the collinear case. In this particular case, $\mathbf{n} = (1, 1, 0)$, the matrix (27) reduces

Due to the invariance of the tensor under interchange of some of its components, the following abbreviation has been applied:

$c_{ijk} \rightarrow c_{\alpha\beta}$
$ij \rightarrow \alpha$
$k \rightarrow \beta$

The components of the tensor are given in the (orthogonal) setting where the symmetry operations of the input are given.

Table of tensor components of the magnetic point group

$c_{\alpha\beta}$	β			
α		1	2	3
	1	$\mathbf{c_{11}}$	$\mathbf{c_{12}}$	0
	2	c_{12}	c_{11}	0
	3	$\mathbf{c_{31}}$	c_{31}	0
	4	0	0	$\mathbf{c_{43}}$
	5	0	0	c_{43}
	6	$\mathbf{c_{61}}$	c_{61}	0

Number of independent coefficients: 5

Figure S2: Symmetry reduced Voigt-like form of the tensor with Jahn symbol ae[V2]V under the MPG formed by the operations of equation (39). The independent coefficients are written in bold.

to,

$$m_{\|\mathbf{n}} = \begin{pmatrix} 0 & 1 & 0 \\ 1 & 0 & 0 \\ 0 & 0 & 1 \end{pmatrix} \tag{41}$$

and the $\mathcal{R}_{m_{\|\mathbf{n}}}$ matrix is,

$$\mathcal{R}_{m_{\|\mathbf{n}}} = \mathbb{I}_3 \otimes \mathbb{I}_3 \otimes m_{\|\mathbf{n}} \tag{42}$$

The matrix that gives the partial reduction of the tensor by the SpPG (before the application of the reduction imposed by the axis $\infty_{\mathbf{n}}$) after the row reduction, and keeping only the non-zero rows of the matrix, the result is shown in table (S4).

The provisional form of the components of the tensor are,

$$(T_1, \ldots, T_{27}) = (c_{111}, c_{113}, c_{331}, c_{333})\mathcal{P}^e \tag{43}$$

with $\mathcal{P}^e$ being the 4×27 matrix shown below the line in table (S4).

Finally, to get the final for of the tensor under the collinear spin group the symmetry restrictions

Table S4: Above the line, the components of a rank-3 tensor are indicated (the indices are aligned vertically to save horizontal space), in the order given by expression (5). Below the line, the table contains the non-zero components of the matrix $\mathcal{P}^e$ in equation (20) that gives the partial symmetry reduction of the tensor with Jahn symbol [V2]M under the SpPG with elements in equation (38) (after the row reduction). To get the final reduction, the additional constraints imposed by the infinite axis must be added.

c	c	c	c	c	c	c	c	c	c	c	c	c	c	c	c	c	c	c	c	c	c	c	c	c	c	c
1	1	1	1	1	1	1	1	1	2	2	2	2	2	2	2	2	2	3	3	3	3	3	3	3	3	3
1	1	1	2	2	2	3	3	3	1	1	1	2	2	2	3	3	3	1	1	1	2	2	2	3	3	3
1	2	3	1	2	3	1	2	3	1	2	3	1	2	3	1	2	3	1	2	3	1	2	3	1	2	3
1	1	0	0	0	0	0	0	0	0	0	0	1	1	0	0	0	0	0	0	0	0	0	0	0	0	0
0	0	1	0	0	0	0	0	0	0	0	0	0	0	1	0	0	0	0	0	0	0	0	0	0	0	0
0	0	0	0	0	0	0	0	0	0	0	0	0	0	0	0	0	0	0	0	0	0	0	0	1	1	0
0	0	0	0	0	0	0	0	0	0	0	0	0	0	0	0	0	0	0	0	0	0	0	0	0	0	1

imposed by the infinite axis must be added. The ω matrix of equation (31) is,

$$\omega = \begin{pmatrix} 0 & 0 & \frac{1}{\sqrt{2}} \\ 0 & 0 & -\frac{1}{\sqrt{2}} \\ -\frac{1}{\sqrt{2}} & \frac{1}{\sqrt{2}} & 0 \end{pmatrix} \tag{44}$$

and the Ω matrix necessary to write the homogeneous linear equation (34) is,

$$\Omega = \frac{1}{\varphi}\left(\mathcal{R}^{\infty} - \mathbb{I}_{3^r}\right) = \mathbb{I}_3 \otimes \mathbb{I}_3 \otimes \omega \tag{45}$$

The linear set of equations (34) is,

$$(c_{111}, c_{113}, c_{331}, c_{333})\mathcal{P}^e\Omega \tag{46}$$

whose general solution is $c_{113} = c_{333} = 0$. The table (S4) transforms thus into table (S5). The

Table S5: Above the line, the components of a rank-3 tensor are indicated (the indices are aligned vertically to save horizontal space), in the Voigt-like notation. Below the line, the table contains the non-zero components of the matrix $\mathcal{P}'^e$ in equation (35). This matrix gives the final symmetry reduction of the tensor with Jahn symbol [V2]M under the SpPG with elements in equation (38) (after the row reduction).

c	c	c	c	c	c	c	c	c	c	c	c	c	c	c	c	c	c
1	1	1	2	2	2	3	3	3	4	4	4	5	5	5	6	6	6
1	2	3	1	2	3	1	2	3	1	2	3	1	2	3	1	2	3
1	1	0	1	1	0	0	0	0	0	0	0	0	0	0	0	0	0
0	0	0	0	0	0	1	1	0	0	0	0	0	0	0	0	0	0

final tensor has only 2 independent parameters, chosen as c_{11} and c_{31} in the Voigt form, and 6 non-zero components, $c_{11} = c_{12} = c_{21} = c_{22}$ and $c_{31} = c_{32}$. Fig. (S3) reproduces *STENSOR*'s third

part of the output.

Due to the invariance of the tensor under interchange of some of its components, the following abbreviation has been applied:

$c_{ijk} \rightarrow c_{\alpha\beta}$
$ij \rightarrow \alpha$
$k \rightarrow \beta$

Table of tensor components of the spin point group

$c_{\alpha\beta}$	β			
α		1	2	3
	1	**c_{11}**	c_{11}	0
	2	c_{11}	c_{11}	0
	3	**c_{31}**	c_{31}	0
	4	0	0	0
	5	0	0	0
	6	0	0	0

Number of independent coefficients: 2

Figure S3: Symmetry reduced Voigt-like form of the tensor with Jahn symbol [V2]M under the SpPG formed by the operations of equation (38). The independent coefficients are written in bold.

References

Bradley, C. J. & Cracknell, A. P. (1972). *The Mathematical Theory of Symmetry in Solids*. Oxford: Clarendon Press.

Brown, P. J., Nunez, V., Tasset, F., Forsyth, J. B. & Radhakrishna, P. (1990). *J. Phys. Condens. Matter*, **2**(47), 9409–9422.

Chen, X., Ren, J., Zhu, Y., Yu, Y., Zhang, A., Liu, P., Li, J., Liu, Y., Li, C. & Liu, Q. (2024). *Phys. Rev. X*. **14**(3), 031038.

Dresselhaus, M. S., Dresselhaus, G. & Jorio, A. (2008). *Group Theory. Application to the Physics of Condensed Matter*. Berlin: Springer-Verlag.

Etxebarria, J., Perez-Mato, J. M., Tasci, E. S. & Elcoro, L. (2025). *Acta Crystallogr. A Found. Adv.* **81**(4), 1–22.

Gallego, S. V., Etxebarria, J., Elcoro, L., Tasci, E. S. & Perez-Mato, J. M. (2019). *Acta Crystallogr. A Found. Adv.* **75**(3), 438–447.

Gallego, S. V., Perez-Mato, J. M., Elcoro, L., Tasci, E. S., Hanson, R. M., Momma, K., Aroyo, M. I. & Madariaga, G. (2016). *J. Appl. Crystallogr.* **49**(5), 1750–1776.

Grimmer, H. (1993). *Acta Crystallogr. A*, **49**(5), 763–771.

Grimmer, H. (2017). *Acta Crystallogr. A Found. Adv.* **73**(4), 333–345.

Hanson, R. M., Prilusky, J., Renjian, Z., Nakane, T. & Sussman, J. L. (2013). *Isr. J. Chem.* **53**(3-4), 207–216.

Jahn, H. A. (1949). *Acta Crystallogr.* **2**(1), 30–33.

Jiang, Y., Song, Z., Zhu, T., Fang, Z., Weng, H., Liu, Z.-X., Yang, J. & Fang, C. (2024). *Phys. Rev. X*. **14**(3), 031039.

Litvin, D. B. (1977). *Acta Crystallogr. A*, **33**(2), 279–287.

Litvin, D. B. (2013). *Magnetic Group Tables: 1- 2- and 3-dimensional magnetic subperiodic groups and space groups.* Chester, England: International Union of Crystallography.

Litvin, D. B. & Opechowski, W. (1974). *Physica*, **76**(3), 538–554.

Liu, P., Li, J., Han, J., Wan, X. & Liu, Q. (2022). *Phys. Rev. X.* **12**, 021016.

Nye, J. F. (1985). *Physical properties of crystals.* Oxford science publications. London, England: Oxford University Press.

Radaelli, P. G. (2024). *Phys. Rev. B.* **110**(21), 214428.

Šmejkal, L., Sinova, J. & Jungwirth, T. (2022*a*). *Phys. Rev. X.* **12**(3), 031042.

Šmejkal, L., Sinova, J. & Jungwirth, T. (2022*b*). *Phys. Rev. X.* **12**(4), 040501.

Spaldin, N. A., Fiebig, M. & Mostovoy, M. (2008). *J. Phys. Condens. Matter*, **20**(43), 434203.

Tomiyasu, K. & Kagomiya, I. (2004). *J. Phys. Soc. Jpn.* **73**(9), 2539–2542.

Watanabe, H., Shinohara, K., Nomoto, T., Togo, A. & Arita, R. (2024). *Phys. Rev. B.* **109**(9), 094438.

Xiao, R.-C., Jin, Y., Zhang, Z.-F., Feng, Z.-H., Shao, D.-F. & Tian, M. (2025). *Comput. Phys. Commun.* **318**(109872), 109872.

Xiao, Z., Zhao, J., Li, Y., Shindou, R. & Song, Z.-D. (2024). *Phys. Rev. X.* **14**(3), 031037.

Yamani, Z., Tun, Z. & Ryan, D. H. (2010). *Can. J. Phys.* **88**(10), 771–797.

Zhu, H., Li, J., Chen, X., Yu, Y. & Liu, Q. (2025). *Nature Communications*, **16**(1), 4882.
